# Systematic drug perturbations on cancer cells reveal diverse exit paths from proliferative state

**DOI:** 10.18632/oncotarget.7294

**Published:** 2016-02-09

**Authors:** Joseph X. Zhou, Zerrin Isik, Caide Xiao, Irit Rubin, Stuart A. Kauffman, Michael Schroeder, Sui Huang

**Affiliations:** ^1^ Institute for Systems Biology, Seattle WA, USA; ^2^ Computer Engineering Department, Dokuz Eylul University, Izmir, Turkey; ^3^ Institute for Biocomplexity and Informatics, University of Calgary, Alberta, Canada; ^4^ Biotechnology Center, TU Dresden, Dresden, Germany

**Keywords:** cancer drug screen, cancer cell differentiation, dynamical system, cell state transition, gene regulatory network

## Abstract

During a cell state transition, cells travel along trajectories in a gene expression state space. This dynamical systems framework complements the traditional concept of molecular pathways that drive cell phenotype switching. To expose the structure that hinders cancer cells from exiting robust proliferative state, we assessed the perturbation capacity of a drug library and identified 16 non-cytotoxic compounds that stimulate MCF7 breast cancer cells to exit from proliferative state to differentiated state. The transcriptome trajectories triggered by these drugs diverged, then converged. Chemical structures and drug targets of these compounds overlapped minimally. However, a network analysis of targeted pathways identified a core signaling pathway - indicating common stress-response and down-regulation of STAT1 before differentiation. This multi-trajectory analysis explores the cells' state transition with a multitude of perturbations in combination with traditional pathway analysis, leading to an encompassing picture of the dynamics of a therapeutically desired cell-state switching.

## INTRODUCTION

Systematic and genome-wide analysis of static molecular signatures of cancer cells and their susceptibility to panels of cancer drugs [1-4] and more recently, of transcriptome dynamics of cancer cells responding to libraries of drugs [5-7] have established encyclopedias of cell lines and their drug response profiles that can be used to predict therapy response and to repurpose drugs [8-10]. However, these analyses were performed without considering the fundamental principles of the state transition dynamics of large molecular regulatory networks that govern cell state transitions.

Any directed change of a cell's phenotypic state, such as the therapeutically desired transition from a proliferative or a stem-like to an apoptotic or a quiescent, differentiated state is driven by the coordinated change of activities at a large number of gene loci across the genome. Because genes influence one another's expression *via* a network of regulatory interactions, genes cannot alter their expression independently, and transcriptomes (which are the measurable proxies for genome-wide gene activation profiles and hence, for cell states) can change only in a highly constrained manner. In the mathematical abstraction of the high-dimensional gene expression state space in which every transcriptome is specified by a point, the network constraints impose a particular structure on transcriptome dynamics - the shift of transcriptomes in state space. Constraints are most prosaically epitomized by “preferred” states: the attractor states that represent the stable, observable cell phenotypes in which all gene regulatory interactions are satisfied [11]. Accordingly, the switch from one stable cell state to another corresponds to a transition from one attractor to another [12, 13] in which the transcriptome moves along a specific trajectory in state space that manifests the state space structure. However, while studies have focused on molecular pathways that actuate a transcriptome change, the structure of the state space around and between attractors of cancer cells is still unexplored.

## RESULTS

### Drug screening and transcriptome measurement

**Figure 1:**
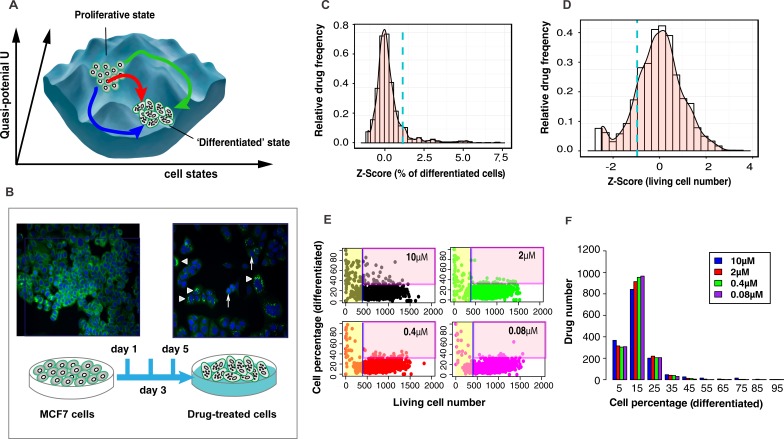
Experimental system and statistics of drug screening results **A.** A structure of state space with multiple transition paths reflects the constraints imposed by gene regulatory network - being depicted as a quasi-potential landscape [30]. **B.** Screening bioassay: lipid-vesicles stained with green fluorescence LIPTOL. Arrow head - undifferentiated cells; triangles - differentiated cells; **C.** Histogram of differentiation efficiency in Z-Score for 1,528 drugs at 10 μM. The selection criteria is: Z-Score > 1.8 (blue dashed line). **D.** Histogram of living cell number in Z-Score. The selection criteria is: Z-Scores > −1. **E.** Scatter plots of all drugs ( = dots) for the effects on viability *vs*. differentiation at day 5. Drugs were defined to be ‘non-toxic’ when living cell number > 410 (Z-Score > −1) and ‘effective’ when differentiated cells > 30% (Z-Score > 1.8). **F.** Histogram of differentiation efficiency (in percentage of differentiated cells) of all drugs at all doses (note: there are about 25% basal (spontaneous) dose-independent differentiation).

To expose the state space constraints that govern the potential exit from the cancerous attractor state and to confirm that differentiation therapy switches cancers to a new attractor state, as proposed more than 40 years ago [14, 15], we screened the Johns Hopkins Chemical Compound Library (JHCCL) of 1,528 FDA approved drugs [16] for compounds with the ability to stimulate MCF7 cells, a metastatic breast cancer cell line successfully used in drug discovery [17], to exit from the default [18] proliferative state to a differentiated state. Trajectories triggered by 16 differentiation-inducing drugs provided a first glimpse on the state space “landscape” (Figure 1A) around the attractor state of cancer cells.

Using high-content-high-throughput (HC/HT) single-cell resolution screening for fluorescently stained milk lipid vesicles in MCF7 cells that mark a non-proliferative state with features of differentiation (Figure 1A, 1B, see experiment details in Material and Methods), we identified 16 non-cytotoxic compounds that converted > 30% of cells into a lipid-vesicle positive state after 5 days (Figure 1C, 1D, 1E; Table S1; confirmation by commercial versions of the compounds in Figure S2). They include antibiotics, psychopharmaceuticals, etc. but no chemotherapeutic agents. The statistical distribution of efficacies (% of lipid-vesicle positive cells) among the 1,528 drugs (Figure 1F) shows the pharmacologically available “perturbation space” of these cells represented by the library. Interestingly, while only 14 drugs had > 75% differentiating activity (e.g., Thiostrepton, as previously reported [19]), a large group of 105 drugs displayed moderate (30-75%) dose-dependent ability to differentiate MCF7 cells. Such intermediate drugs, usually discarded in industrial drug screening, reflect cell population heterogeneity in susceptibility [20] and can be exploited to map the state space constraints.

To characterize the high-dimensional gene expression trajectories along which cells move to the differentiated state, we measured transcriptomes of the MCF7 cells at Day 1, Day 3, Day 5 after incubation with each of the 16 selected compounds using DNA microarrays. For an error model for sample-to-sample variation, we measured 14 replicates of untreated cells as the gene expression reference (see Material and Methods). A subset of 2,013 genes that were differentially expressed (based on Significance Analysis of Microarrays [21]) between control and treated samples in at least one time point were used for subsequent analysis.

### The divergence-convergence patterns of cell differentiation trajectories

**Figure 2:**
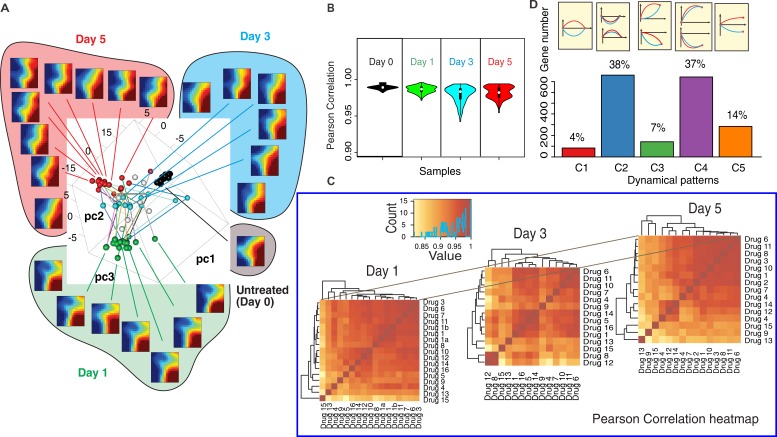
The transcriptomes of 16 effective drugs diverge and converge **A.** Principle Component Analysis (PCA) of time-courses of the transcriptomes of MCF7 cells responding to the 16 effective drugs. Each transcriptome sample is plotted as a sphere ball in the spanned space of the top three PCA components (black - Day 0; green - Day 1; blue - Day 3, red - Day 5). The transcriptomes of the selected samples were plotted in the self-organizing maps (heatmap) using Gene Expression Dynamics Inspector (GEDI) - showing that gene expression profiles are similar in Day 1 and Day 5 while they are quite different in Day 3. **B.** Violin plot of Pearson correlation coefficients quantify the dispersion of the transcriptomes in time. **C.** Heatmaps of Pearson correlation coefficients show the trend of divergence/convergence of the transcriptomes. (see drug No.-drug name mapping at Suppl. Table S1-1) **D.** The frequencies of various categories of gene expression patterns following perturbations with different drugs (blue and red curve represent response to two distinct drugs) for the 5 categories: C1-C5.

To visually represent the global dynamics, the transcriptomes of all 16×3 samples (drugs × time points) and untreated controls were mapped into a 3D-state space using principal component analysis (PCA) (Figure 2A). For selected samples we displayed self-organizing maps using Gene Expression Dynamics Inspector (GEDI) [22]. This global view (Figure 2A GEDI heatmaps) reveals that transcriptome changes in response to the drugs were remarkably similar at Day 1 and Day 5. However, responses diversified at Day 1 (spread of green spheres in Figure 2A) compared to controls (black spheres, day 0). Diversity was maximal at Day 3 (blue spheres) but transcriptomes converged at Day 5 although they did not reach the same compactness as before treatment.

This divergence-convergence of trajectories is expected if the drugs act on distinct biochemically pathways but move the cells to the same (or similar) attractor state(s) [23]. To quantify the transient dispersion at Day3, we computed the Pearson correlation coefficients between all pairs of transcriptomes. The mean sample-sample correlation was both lowest and had widest spread at Day 3 (Figure 2B) -consistent with diversification of transcriptomes. Statistical analysis of the 14 controls and random gene-subsampling show that the divergence did not reflect measurement noise and that the divergence-convergence pattern was robust (Figure S6). Thus, drug-induced exit from the proliferative state did not follow a single trajectory in gene expression state space. However, the heatmaps of correlation coefficients between all transcriptomes and hierarchical clustering (Figure 2C) revealed large cell clusters at Day 1 and Day 5 which broke into many smaller clusters at Day 3, suggesting that trajectories did not diversify into a continuum but were bundled into subgroups, manifesting constraints of the transient dynamics.

The diversity of state transition trajectories offers a new perspective for classifying genes beyond traditional “differential expression” (up- or down-regulation) and gene clusters. We divided genes according to their multi-trajectory behavior into five categories C1-C5 with respect to the relative trajectory courses shown in Figure 2D (see quantitative criteria in Methods). Both groups of genes that either did not exhibit a net change of expression between Day 0 and Day 5 (C1+C2), or changed significantly (C3+C4) contained genes that exhibited significant transient divergence before convergence (C1+C3). These transiently disparate genes underlie the divergence-convergence course of trajectories (Figure 2A) and may have a role in destabilizing the original cell state to overcome the “energy barrier” between the cell states [13]. Their early disparity indicates that various drugs trigger distinct transient destabilizations, hence distinct exit paths. The most prominent genes of these transient responses belong to groups functionally linked to apoptosis, cell stress and inflammatory response (Table S3, S4). By contrast, most genes displaying net differential expression in untreated *vs*. differentiated cells (C4) changed in a concordant way, pointing to a core set of genes possibly involved in guiding the state change towards the differentiated state as opposed to the genes driving the exit from the proliferative state.

### The diversity of drugs that share the similar phenotype effect

**Figure 3:**
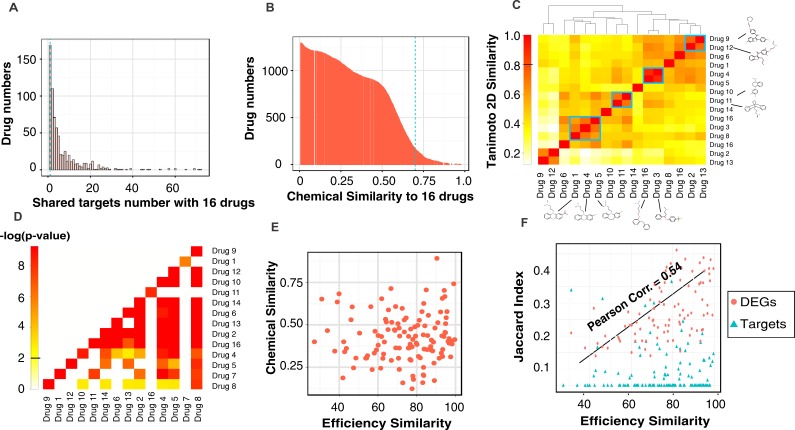
The statistics of drug characteristics and the common features of the 16 effective drugs Histograms of 1,528 drugs that **A.** share targets and **B.** have structural similarity with the 16 effective drugs. **C.** The heatmap showed the pairwise 2D-Tanimoto Chemical Similarity Scores (TS) among the 16 drugs. Overall similarity is low (TS < 0.7); blue rectangles mark the most similar pairs with their 2D structures displayed. **D.** The heatmap of common targets. Color represents log(*P*-value) of target sharing between any pair of the 16 drugs by chance. **E.** There is no correlation between the chemical similarity and drug efficiency of 16 effective drugs (Pearson Correlation Coefficient (PC) = 0.035). **F.** There is no correlation between the shared drug targets and efficiency similarity of 16 drugs while there exists a moderate correlation (PC = 0.54) between shared differentially expressed genes (DEGs) and efficiency similarity. (Target sharing between any drug pair is calculated by Jaccard Index (JI), see Material and Methods).

The dynamical systems view of trajectories converging to attractors explain why biochemically distinct perturbations can elicit a common, specific phenotype [15] but does not exclude a common underlying molecular pathway. We used our 16 differentiation-inducing drugs to expose possible common deterministic pathways using the STITCH drug target database [24]. As baseline, we found that 25% of compounds (*n* = 888) in the JHCCL library share at most one drug target with any of our 16 effective drugs (Figure 3A) and have low chemical similarity with any of these 16 compounds (Tanimoto-2D-similarity (TS) score < 0.7 with *p-*value 0.006 (Figure 3B). By contrast, some of the 16 effective drugs display partial chemical similarity among themselves (Figure 3C); the most similar pair was *Fluoxetine* (drug *4,* Figure 3C) and *Bifemelane* (drug *5*) with TS = 0.89. 47% of all pairs among the 16 drugs share at least 1 common target (Figure 3D *P*-value < 0.01; for significance estimation see Material and Methods). However, the similarity in efficacy (% of cells differentiated) did not significantly correlate with chemical similarity (Figure 3E) nor with number of shared targets (Figure 3F, blue triangles) but moderately (Pearson Correlation Coefficient = 0.54) with the net transcriptome.

### Common affected paths network (CAP-Net) analysis

**Figure 4:**
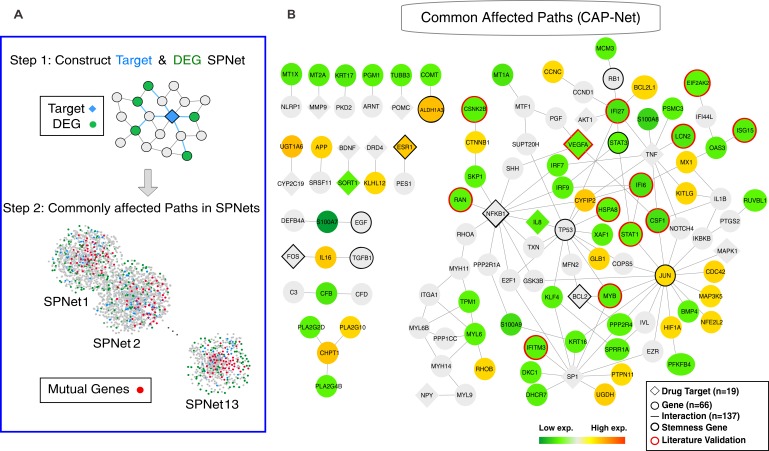
Commonly affected paths (CAP-Net) analysis **A.** The principle of CAP-Net analysis. The shortest paths were calculated between the drug targets and the differentially expressed genes (DEG). The common pathways were found by the pathway overlap among the target-DEG pairs and the different drugs. **B.** CAP-Net showed the commonly affected downstream pathways during MCF7 differentiation (Day 5). Diamonds and circles, respectively, represent the targets and the differentially expressed genes. The color of a node represents the average expression level of the corresponding gene.

**Figure 5:**
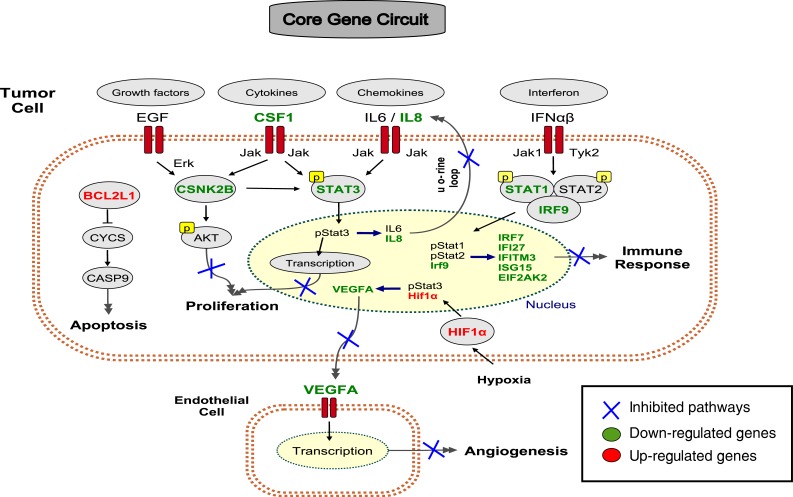
A core gene circuit of genes putatively involved in drug-induced MCF7 cell differentiation We inferred a core gene circuit from the CAP-Net analysis. The immune response is down-regulated through *Interferon* pathway and *Jak*-*STAT1* pathway. The proliferation pathway is down-regulated through growth factors *EGF*, Cytokines *CSF1*, and Chemokines *IL6/IL8* through *Jak*-*STAT3* pathway. The apoptosis pathway is activated through *BCL2L1*. Angiogenesis pathway is down-regulated through *VEFGA*.

Despite the low number of common drug targets, we asked if the 16 effective compounds still influence common downstream effectors in view of the robust core of concordant changes in gene expression that define the transition trajectory (Figure 2D category C4 genes). We developed a shortest-path-based method - Commonly Affected Paths Network (CAP-Net) - to identify common downstream pathways that may contribute to the same phenotypic drug effect (see Material and Methods). CAP-Net uses two databases, STITCH (known and predicted drug targets) and STRING (known and predicted protein interactions) [25], to compute plausible pathways linking drug-targets to genes whose expression was significantly changed by the drugs. For each drug, CAP-Net computes the shortest paths on the protein-interaction network (extracted from STRING) between all pairs of immediate targets to the differentially expressed genes (Step 1 in Figure 4A). We then assembled all edges that appear in the shortest paths between at least two different drugs (Step 2 in Figure 4A). Using the overlapping transcriptomes of the differentiated state (Day 5), CAP-Net analysis led to a set of common molecular pathways shared by the effective compounds (Figure 4B for Day 5 and Figure S7 for Day 1) as well as a core circuit (see Figure 5). They include interferon family proteins (*IFI6, IFI27, IFIT1* and *IFITM3* etc. more in Suppl.) and, more importantly, *STAT1* [26] and *STAT3* which were significantly down-regulated and would have been missed in traditional transcriptome analysis.

## DISCUSSION

The molecular mechanism of a cell state transition is usually revealed by identifying the differentially expressed genes from the transcriptomes at two conditions - GO term analysis is used to find their biological functions while the knock-out or over-/down-expression experiments to validate the key genes' functions. From multiple time courses of transition transcriptomes induced by different drugs, our analysis of gene transition patterns shows that the traditional approach is generally correct: a dominating trajectory in gene expression state space is shared by most drugs. However, the exit paths from the proliferation state vary from drug to drug. The transiently expressed genes and diverging-converging genes strongly influence the cell state transition - although we usually neglect them.

We found that MCF7 cells exit proliferation states in several distinctive trajectories after being stimulated by different drugs. Among the genes which significantly changed expression levels, about 10% of them diverged at first and converge later (Figure 2D). It means that cells are destabilized by drug stress, then move to different directions and may fall into the same cell state which are defined by the gene-gene interactions from gene regulatory network. The destabilization mechanism also explains why many nonspecific drugs induced MCF7 cells differentiation in low efficiency (Figure 1F). These drugs destabilize the cancer cell state but lack of stimulus to guide the cells to differentiated state. The discovery implied a new direction of cancer drug development: rather than identifying one drug which cause cell transition in a well-defined pathway, we can use multiple drugs to destabilize the proliferation state of cancer and induce cells to exit in various ways.

Although gene expression time course is usually avoided in transcriptome analysis of cell state transition. It is inadequate to identify the molecular mechanism of cell state transition by examining the differentially expressed genes only. Our analysis showed that about 11% of genes are transiently expressed during the cell state transition, which are generally neglected (Figure 2D). GO term analysis showed that these genes involve in apoptosis, cell stress/defense response, inflammatory response, cell replication, DNA translation, macromolecular complex assembly, mitochondrial activities etc. They influence the cell state transition by destabilizing the original cellular state and triggering the following cell state changes.

Even 16 drugs all lead MCF7 cells exit proliferation state, they do not bind to the same targets. According to Tanimoto 2D similarity score (cutoff = 0.8), most drugs do not share the same chemical structure (Figure 3C) and their chemical similarity do not correlate to drug efficacy (Figure 3E). These drugs share some targets, which do not correlate to the drug efficacy. Why do these drugs have similar effects when they do not share similar chemical structures and not share common causal drug targets? From our Common Affected Pathways (CAP-Net) analysis, most drugs have targets in a common pathway to trigger the MCF7 differentiation. They bind to different targets, but these targets locate in the same pathway of exiting proliferation state. A moderate correlation is found between the drug efficacy and the common differentially expressed genes triggered by drugs (Figure 3F). It means that drugs with common effects can have different structures and bind to distinct drug targets. If we can identify the causal pathway of the drug, the similar effects of drugs can be found by identifying all possible drug targets along its effective pathways.

In conclusion, we used a drug library not only to identify new therapeutics but also as a tool to apply a diversity of perturbations to probe the “landscape” of gene expression state space that determines the differentiation paths available for cancer cells to exit the malignant stem-like state. Although the partial response to drugs likely stem from cell population heterogeneity and individual cells may utilize distinct single-cell trajectories, the multi-perturbed transcriptome time courses of the whole cell population collectively revealed that the drugs trigger the transition of cancer cells out of the proliferative state *via* various paths. This analysis opens a new perspective for identifying genes in causative pathways of malignancy by combining a dynamics systems framework with traditional transcriptome and pathway analysis, which might not have been revealed by simple differentially expressed gene analysis.

## MATERIALS AND METHODS

All computational analyses were performed using R, unless otherwise indicated.

### Cell culture and drug screen

### MCF7 cell culture

MCF7 cells, a breast cancer cell line of the luminal type (Figure S1), were derived from a single progenitor cell and were grown in DMEM (Dulbecco's Modified Eagle Medium) with 4.5 g/L glucose and L-Glutamine, without sodium pyruvate (DMEM, Corning), supplemented with 10% fetal bovine serum (FBS, Atlanta Biologicals), penicillin (100 U/mL, Gibco) and streptomycin (100 mg/mL, Gibco), and insulin at 10 μg/ml (Insulin solution from bovine pancreas, Sigma). The cells were grown to 80% confluency. They were passaged approximately every 3-4 days: washed with 1XPBS (PBS, pH 7.4, Gibco), trypsinized (Trypsin-EDTA, Gibco) and split at about 1:4 ratio).

### High-content-high-throughput (HC/HT) drug screening for MCF7 cell differentiation based on automated image analysis of fluorescently (LipidTOX) stained lipid vesicles

MCF7 cell suspension (100 μL) was plated into each well of 96-well plates at day 1 using a liquid handling robot (Beckman Coulter Biomek FX). Each drug solution (dissolved 100 μL) at the concentrations 0.08 μM, 0.4 μM, 2 μM and 10 μM in a separate VWR square deep-well 96-well titer plate was transferred to the cell culture by the robot. The liquid volume in each cell culture well was 200 μL.

After MCF7 cells had been subjected to drugs for five days, cell were fixed and stained using the robot: Cells in each well were washed in PBS and fixed with 3.8% formaldehyde (in 100 μL PBS) for 20-30 minutes. Cells were washed with 160 μL PBS and stained with 50 μL LipidTOX Green phospholipid stain (Invitrogen) diluted 1:1000 in PBS. After 30 minutes incubation another 50 μL 4′,6-diamidino-2-phenylindole at 1:10000 in PBS (DAPI, Invitrogen) was added to the cells for another 30 minutes to strain the cell nuclei and washed with 160 μL PBS. The wash was replaced with a final 160 μL PBS and scanned by an IN Cell 1000 image analyzer (GE Healthcare Life Sciences). Four random fields per well were photographed (with 20X objective) at both wave length (for LipidTOX and DAPI). The percentage of positive cells (per living cell) were scored using the IN Cell 1000 workstation software (v. 3.4) after segmentation and non-apoptotic cell count based on DAPI; a cell with at least one lipid droplet was defined as a positive cell. The computationally determined percentage of positive cells correlated well with visual determination (not shown).

### Drug screening criteria

The drugs considered “effective” were chosen using two criteria, differentiation efficiency and toxicity: (1) the LipidTOX dye positive cell percentage after 5 days' treatment of drugs at 10 μM Z-Score ≥ 1 (Figure 1C) (2) Toxicity Z-Score ≥ −1 was used to exclude drugs that were toxic. Toxicity was defined as the number of surviving cells (count live cells) at 10 μM drug (Figure 1D). All 16 drugs passed these two criteria.

### List of 16 drugs, cell differentiation efficiencies and their dose-response curves

All 16 drugs are listed in Table S1-1 and dose-response curves are shown in Figure S2.

### DNA microarrays (Illumina HumanHT-12 BeadChip)

For gene expression profiling, MCF7 cells were cultured in 150mm dishes and treated 1/5/10 μM of each of the 16 drugs (see details in Tables S1-2). 14 plates of cells were left untreated as control samples. Cells were collected after 1, 3 and 5 days of drug treatment in RNeasy (Qiagen) lysis buffer and RNA was isolated according to the manufacture's protocol and sent to Vancouver Prostate Center for transcript profiling using the Illumina HumanHT-12 v4 Expression BeadChip. The accesion number for the expression data is GEO: GSE74281.

### Gene expression profile analysis

### Filtering out the background noise of gene expression

Illumina BeadChip has an internal background control of gene expression, thus gene expression significantly below the background threshold was considered as unreliable signal (detection *p*-value > 0.05). Since the gene expressions are measured as a time course, the criteria of a gene of being significantly expressed was: detection P-value ≤ 0.05 for at least one day in the entire whole time series.

### Error model and transcriptomes

The error model used is shown in Figure S3 based on the 14 replicates of untreated samples. Self-organizing maps (SOM) were generated with the gene expression dynamics inspector (GEDI) software [22]. The transcriptomes for samples of cells treated with selected drugs at different time points are illustrated as GEDI maps. Each GEDI map represents a microarray measurement or a transcriptome of an RNA sample ( = cell culture dish collected), that is, of a drug and a time point. (See Suppl. Figure S4 for entire set of GEDI maps). In GEDI maps the more similarly two genes are expressed across all samples, the closer to each other are they placed on the 2D grid of each GEDI map by the SOM. Each pixel in the GEDI map (grid element) represents a mini-cluster of highly similarly behaving genes. The pixel at the same position in each map represents the same (set of) genes; The color of each pixel represents the average of gene expression level of that set. Thus, global patterns in the GEDI maps give an intuitive notion of the entire transcriptome and allow for visual (“Gestalt”) comparison of the samples. The GEDI heatmap shows the similarities of 16 drug-treated MCF7 differentially expressed genes at different time points (Figure S4).

### The classification of dynamic patterns of gene expression during transition

(1) Criterion for differentially expressed genes upon differentiation (“up/down-regulated genes”) We defined the gene expression levels of one sample as *x* = (*x_1_*, *x_2_*,.., *x_i_*, .., *x_n_*), *n* = total number of genes. Then we calculated the averages of gene expression $\bar{X^{0}}=({\bar{x}}_{1}^{0},\;{\bar{x}}_{2}^{0},\;\cdots \;,{\bar{x}}_{i}^{0},\;\cdots {\bar{x}}_{n}^{0})$ and the standard deviations $\sigma_{X^{0}}=(\sigma_{x_{1}^{0}},\;\sigma_{x_{2}^{0}},\;\cdots \;\sigma_{x_{i}^{0}},\;\cdots ,\;\sigma_{x_{n}^{0}})$ of the gene expressions of 14 untreated MCF7 cell samples. A gene *i* was considered to be “up/down-regulated” if its expression level in a given drug-treated sample *x_i_* satisfied:

$$|x_{i}-{\bar{x}}_{i}^{0}|\;>3\sigma_{x_{i}^{o}}$$

(2) Criterion for defining “divergent-convergent” genes

The availability of multiple drug-induced trajectories (treatment *X* time point) of the cell state affords an important unique perspective to characterize individual genes. In order to compare the gene expression differences caused by different drugs, we calculated the difference *d_i_^m^* of the expression level of gene *x_i_* in the *m* th pair of drugs (total pair number is $M=\frac{16\times 15}{2}=120$ pairs) for a given time point (Day 1, 3 5). If a gene *x_i_* displays the “divergent-convergent” pattern, the average differences *d_i_^m^* of the expression level of gene *x_i_* of all pairs of the drugs at either Day 1 or Day 3 should be bigger than three times the standard deviations of the untreated replicates $3\sigma_{X^{0}}$ while those at Day 5 should be smaller than $3\sigma_{X^{0}}$. The condition for the “divergent-convergent” pattern of a gene *x_i_* is met if the first or the second condition and the third condition are satisfied:

$$\begin{array}{c} \frac{1}{M}\sum_{m=1}^{M}d_{i}^{m}{|}_{\text{day }1}>3\sigma_{x_{i}^{o}}\\ \frac{1}{M}\sum_{m=1}^{M}d_{i}^{m}{|}_{\text{day }3}>3\sigma_{x_{i}^{o}}\\ \frac{1}{M}\sum_{m=1}^{M}d_{i}^{m}{|}_{\text{day }5}<3\sigma_{x_{i}^{o}} \end{array}$$

### Modified pearson correlation coefficients of samples

We used the Pearson correlation coefficient to quantify the difference between the transcriptomes of two samples *X*(*t_i_*), *X*(*t_o_*) at two time points (*X*= a vector of *n* components representing the distinct gene expressions). In “standard” Pearson correlation coefficient *r*(*X*(*t_i_*); *X*(*t_o_*), a deviation (*x_i_*-*x_i_*) is calculated between a gene expression value *x_i_* and the mean expression value $\bar{x}$ of all genes in this sample. Here we used a modified form in which the deviation is between a gene expression value *x_i_* and its own mean in all the samples along the time course for one drug [27]. This formula measures the temporal correlation of transcriptome deviations from their average values over time.

To evaluate the statistical robustness of the divergent-convergent pattern, we plotted the distribution of 100 “modified” Pearson correlation coefficients *r* between all pairs of samples treated by the 16 drugs at Day 1, Day 3 and Day 5, *vs*. untreated samples, calculated from a set of 500 randomly-chosen genes (Figure S5). Indeed, drug-specific transcriptomes at Day 1 and day 5 showed a more similar distribution of *r* than the untreated samples (with the exception of treatment with *Desloratadine, Flunisolide, Guanfacine and Maprotiline*) whereas transcriptomes at day 3 had a distribution of correlation values that was remarkably distinct from that of other sample points. This finding further corroborated the finding that the drug-elicited transcriptomes at day 1 and day 5 are more similar to the untreated controls than the transcriptomes at day 3 are.

### Identify the differentially expressed genes and gene sets during MCF7 differentiation

Statistical significance analysis (SAM) of the differentially expressed genes was performed using the SAM 3.0 program [21] comparing the microarray samples in three settings: the transcriptomes of drug-treated samples at day 0 (non-treated) *vs*. day 1 *vs*. day 5, the transcriptomes at day 0 *vs*. day 1 and the transcriptomes at day 0 *vs*. day 5. Differentially expressed genes list from SAM analysis are shown in Table S2. We exploited the fact that day 1 and day 5 transcriptomes clustered closely together, forming two distinct clusters separated from the untreated samples to use standard multi-class SAM to identify gene expression changes common to most drugs (Figure S5A shows the top 138 differentially expressed genes. Gene names are listed in Suppl. Table S2). The differential expressions in day 1 samples (Day 1 *vs* Day 0) across all drug treatments (Figure S5B) showed a common (transient) early-response in which the most upregulated genes were *CYP1A1*, *CYP1B1*, *TGFB* etc. and the most down-regulated genes included *KRT13, IFI6, IFI27, IFIT1* etc. By contrast, differential expression in day 5 samples (Day 5 *vs* Day 0) (Figure S5C) reflected the net state displacement. In the gene expression space, the most down-regulated genes included cytokine *CSF1*, and again, *IFI6/IFI27*, as well as *S100A7* and the most up-regulated genes were *UGT1A6, GRHL3, DNaJB14* and *MYLIP* etc.

While SAM identified significantly differentially expressed genes, we used GSEA algorithm [28] to identify the curated pathways that influence the cell state transition (27) (Suppl. Table S6). The differentially expressed gene sets were analyzed using the GSEA between the microarray samples day 0 *vs*. day 1 and day 0 *vs*. day 5 (shown in Table S3). After 1 day of drug treatment, a prominent response was the expression of genes involved in xenobiotic metabolism - the P450 oxidation system (*CYP1, CYP2, CYP3*), membrane pumps (*ABCB1,4-10, ABCC1-13, ABCD1-3* and *ABCA1-17*), and stress response genes (*RSPOx, CXCL1-7, CXCR1-7, CCL2,3,5*). Thus, despite screening against drug cytotoxicity - which induces defense systems in many cancer cells (28-30), cells still perceived the “differentiating” stimulus by the chemical molecules as xenobiotic attack. Biomarkers of differentiated epithelial breast cells, such as integrin3 and of the ductal cell signatures, were also up-regulated (31, 32). Consistent with the SAM analysis, the expression of immune response genes, notably genes involved in interferon signaling and other cytokine pathways, in particular, *IL12*, were down-regulated. Finally, some generic stemness cell signatures were up-regulated at day 1, which is consistent with cell-stress response (33, 34). Later, in line with the differentiation of MCF7 cells (a luminal type breast cancer), the basal type signatures were down-regulated relative to luminal cell signatures (Suppl. Table S5) (35). At the terminal time point Day 5 (Table S6), the stress and detoxification response of day 1 had subsided but genes of the ductal signatures, steroid hormone synthesis and *IGF1* pathway, were up-regulated; Genes of interferon and cytokine signaling remained low. The expression of genes of the milk-secreting prolactin pathway further increased at day 5 compared to day 1 while the activities of various proliferative pathways decreased.

Taken together, GSEA identified the pathway changes generically associated with the transition to the differentiated state. The transient induction at day 1 of detoxification, stress-response and stemness genes by these drugs suggested that despite screening for non-toxic compounds, cells still experienced cellular stress. The characteristic down-regulation of immune response genes of the *IFN* pathway was not anticipated and its significance requires further studies.

### Drug structures, target and CAP-NET analysis

### Chemical similarity

The chemical similarity of two chemical compounds is computed using Tanimoto Score [29](TS, a value between [0, 1]). It is calculated by using the 2D structure fingerprints:

TS=XY/(X+Y−XY)

where X and Y are the count of bits set in fingerprint X and Y respectively. XY is the count of bits set after bit-wise “AND” operation of fingerprints X and Y. A Tanimoto score of 0.9 or greater is assumed to be statistically very similar.

### Target protein identification

Drug targets are extracted from the drug target database STITCH [24] (version 3.1, accessed at February 2013) online database. This database provides the known drug targets, which are curated from different online databases. In order to limit false positive target assignments, only the high confident drug-target pairs (confidence score > = 0.8) were selected to derive the known targets. This threshold was selected based on the compound-target score distribution in the STITCH database, which contains more than 1.2 million drug-target interactions and ∼150,000 (12.5%) of them have a 0.8 or above confidence score.

The null hypothesis assumes that any randomly chosen drug pair shares the same targets with one pair of 16 drugs. We randomly choose one drug pair out of 1,528 drugs for 10,000 times and check if it has the same common targets with one pair of 16 drugs. In this way, a P-value for sharing the same targets is calculated for each pair of 16 drugs.

### Efficiency similarity

The Efficiency Similarity, *Eff-Sim(a,b)* shows the efficiency similarity between drug *a* and *b*. The individual efficiency scores are converted into a similarity metric:

Eff-Sim(a,b)=100−|E(a)−E(b)|

where *E(a)* and *E(b)* are individual efficiency values of drug *a* and drug *b*, respectively. Their absolute difference is subtracted from 100 to convert this value into a similarity score. If *Eff-Sim(a,b)* is high, two drugs have very similar efficiency scores, or vice versa.

### Jaccard index

The common members of two sets, A and B, are compared by Jaccard Index, i.e. the size of the intersection of A and B sets is divided by the size of their union.

JI=|A∩B|/|A∪B|

### CAP-Net analysis

In order to find out the pathways which cause cancer cell differentiation, we extracted the paths that connect drug targets and corresponding differentially expressed genes (the genes that are 1.5 folds up-/down-regulated between drug-treated and control samples). The topology is obtained from a protein-protein interaction network STRING [25] (version 9.0, accessed at March 2013) which is an integrated homospaiens protein interaction database. A shortest path algorithm is applied during the extraction of the causal paths between drug targets and differentially expressed genes. All possible shortest paths create a shortest path network (SPNet) for each drug. After constructing SPNets of all drugs, the common paths shared by these networks are identified using graph intersection process. If the nodes A and B are found in both SPNet_1_ and SPNet_2_, and if they are connected *via* the same edge in the two networks, then such a connection is called a common path. This operation is performed for all SPNet simultaneously (see Figure 4A), which led to the Common Affected Pathway-Network (CAP-Net) for all drugs. This analysis was applied on various differentially expressed gene sets (i.e. Day 1, Day 3 or Day 5 samples).

The statistical significance of the CAP-Net was calculated using the null model in which we estimated how often any path of CAP-Net can be found in a randomly selected shortest path. All paths in the CAP-Net are statistically significant with a p-value ≤ 0.015. The genes identified by CAP-Net analysis (both Day 1 and Day 5) as the candidates causing MCF7 cell differentiation are listed in Table S7. CAP-Net analysis for drug response transcriptomes at Day 1 is shown in Figure S7. We also infer the gene regulatory circuit for MCF7 cell state transition (Figure 5).

## SUPPLEMENTARY MATERIAL FIGURES AND TABLES


