# Supplementary material for: Systematic drug perturbations on cancer cells reveal diverse exit paths from proliferative state
Source: Oncotarget. 2016 Feb 9;7(7):7415–25. doi: 10.18632/oncotarget.7294 (PMC4884928; doi:10.18632/oncotarget.7294)
Supplement: Supplementary file 1 [file oncotarget-07-7415-s001.pdf]

Systematic drug perturbations on cancer cells reveal diverse exit paths from proliferative state

Supplementary Material

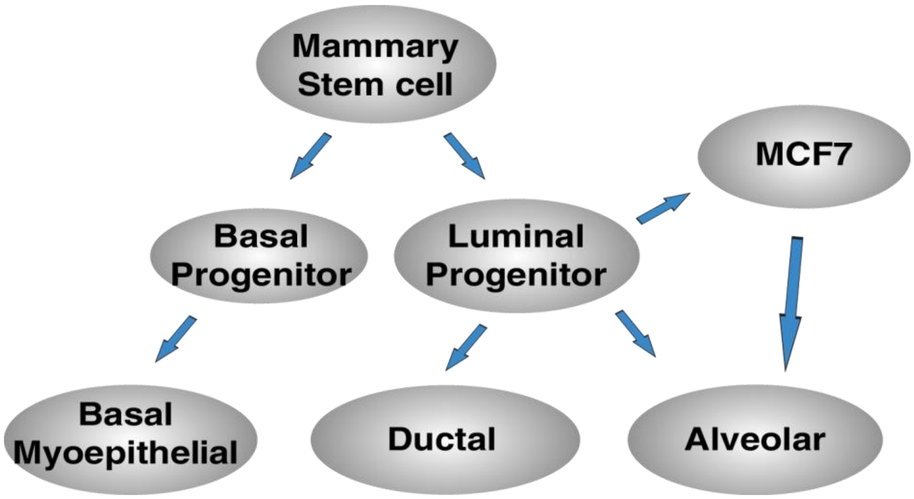

Figure S1. The breast cell development tree and the schema of differentiation therapy which induce breast cancer cells MCF7 to differentiated state.

Mammary stem cells differentiate to Basal progenitors, which differentiate to Basal Myoepithelial; They also differentiate to Luminal progenitors, which differentiate to Ductal and milk-secreting Alveolar cells. MCF7 cells derived from breast Luminal cells are induced to Alveola-like mature state through the selected drugs.

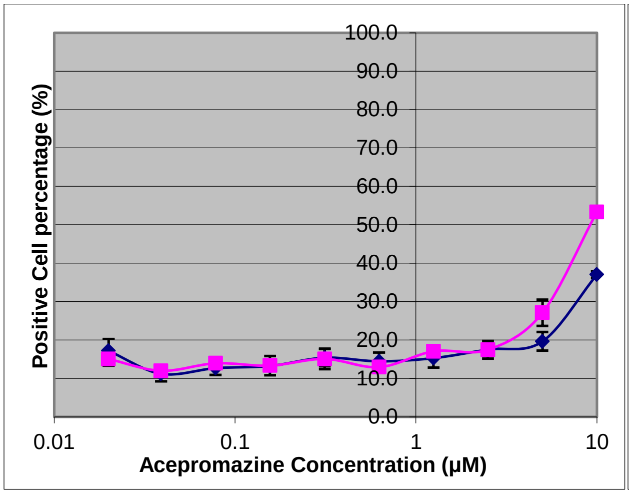

Positive Cell percentage (%)
100.0
90.0
80.0
70.0
60.0
50.0
40.0
30.0
20.0
10.0
0.0
0.01
0.1
1
10
Acepromazine Concentration (μM)

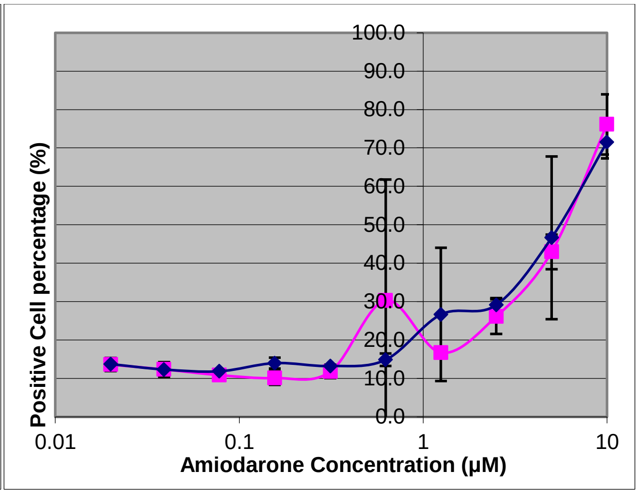

Positive Cell percentage (%)
100.0
90.0
80.0
70.0
60.0
50.0
40.0
30.0
20.0
10.0
0.0
0.01
0.1
1
10
Amiodarone Concentration (μM)

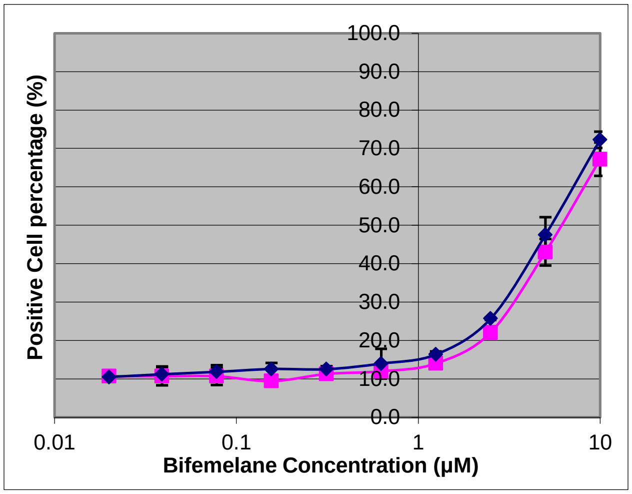

Positive Cell percentage (%)
100.0
90.0
80.0
70.0
60.0
50.0
40.0
30.0
20.0
10.0
0.0
0.01
0.1
1
10
Bifemelane Concentration (μM)

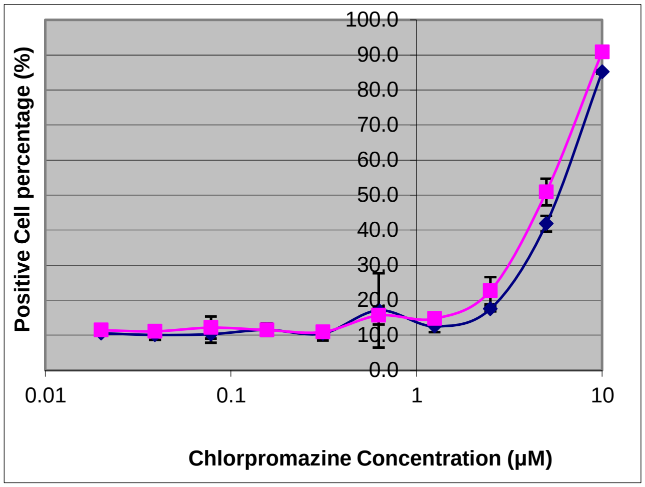

Positive Cell percentage (%)
100.0
90.0
80.0
70.0
60.0
50.0
40.0
30.0
20.0
10.0
0.0
0.01
0.1
1
10
Chlorpromazine Concentration (μM)

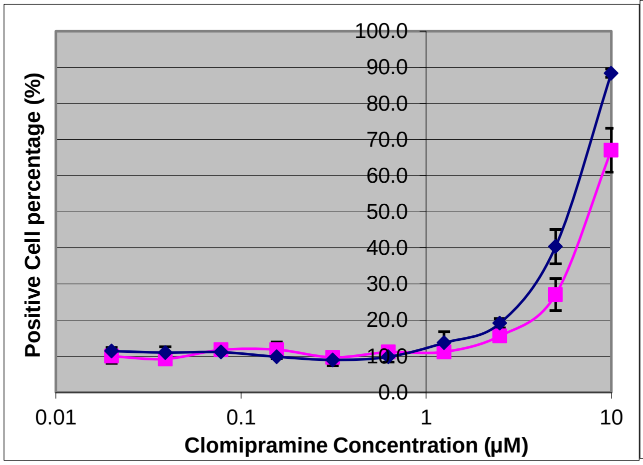

Positive Cell percentage (%)
100.0
90.0
80.0
70.0
60.0
50.0
40.0
30.0
20.0
10.0
0.0
0.01
0.1
1
10
Clomipramine Concentration (μM)

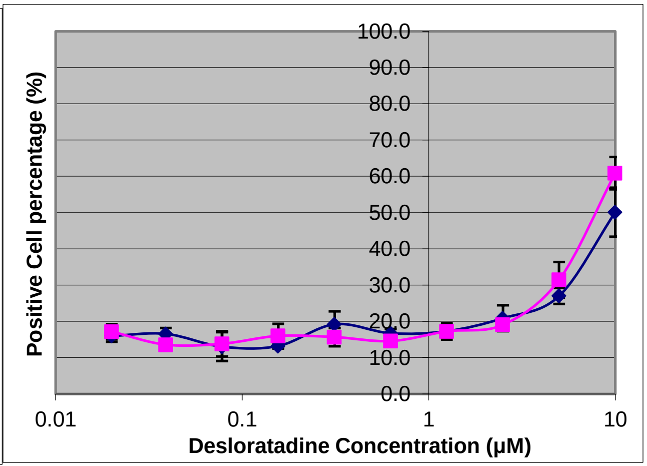

Positive Cell percentage (%)
100.0
90.0
80.0
70.0
60.0
50.0
40.0
30.0
20.0
10.0
0.0
0.01
0.1
1
10
Desloratadine Concentration (μM)

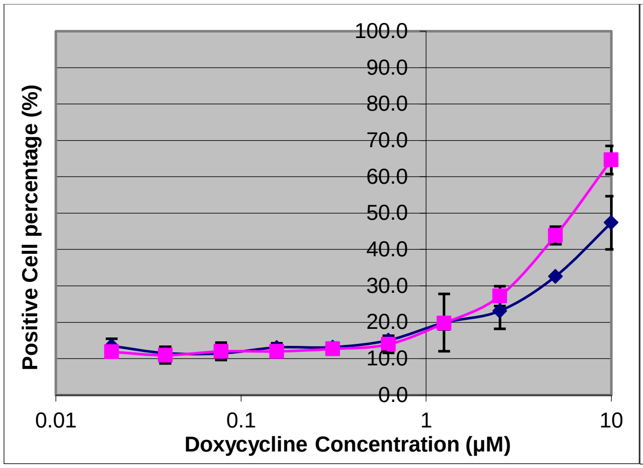
Positive Cell percentage (%)
100.0
90.0
80.0
70.0
60.0
50.0
40.0
30.0
20.0
10.0
0.0
0.01
0.1
1
10
Doxycycline Concentration (μM)

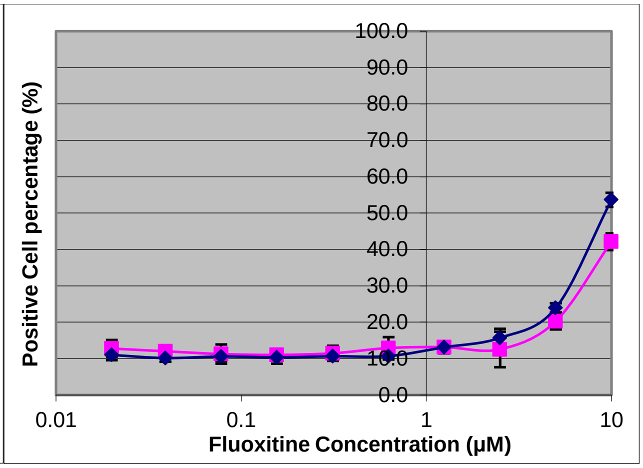
Positive Cell percentage (%)
100.0
90.0
80.0
70.0
60.0
50.0
40.0
30.0
20.0
10.0
0.0
0.01
0.1
1
10
Fluoxitine Concentration (μM)

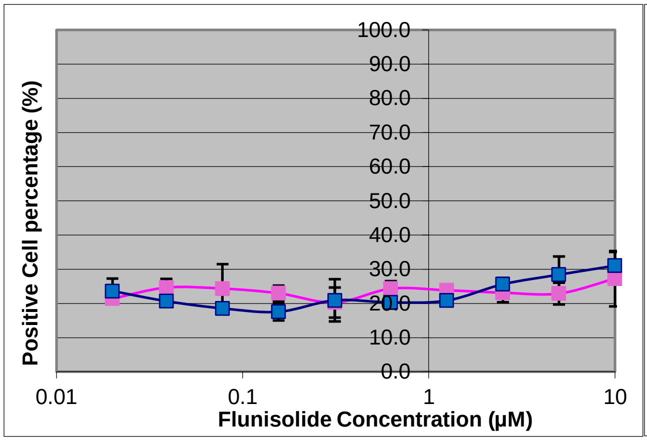
Positive Cell percentage (%)
100.0
90.0
80.0
70.0
60.0
50.0
40.0
30.0
20.0
10.0
0.0
0.01
0.1
1
10
Flunisolide Concentration (μM)

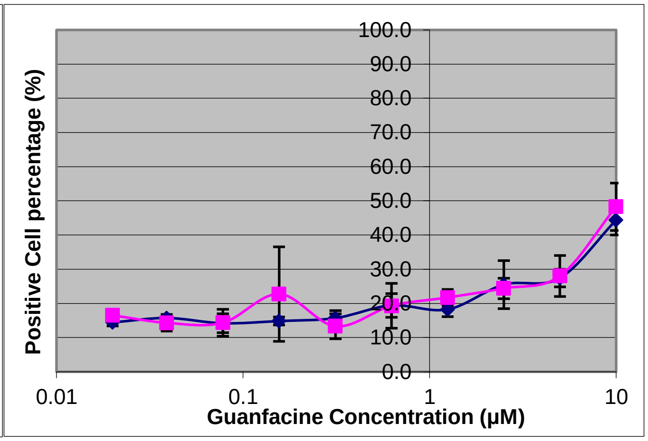
Positive Cell percentage (%)
100.0
90.0
80.0
70.0
60.0
50.0
40.0
30.0
20.0
10.0
0.0
0.01
0.1
1
10
Guanfacine Concentration (μM)

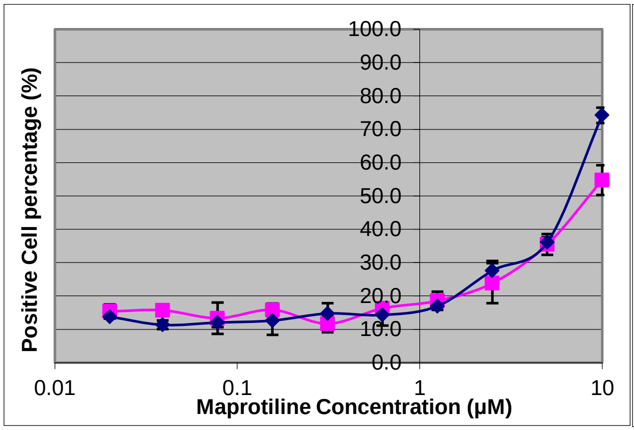
Positive Cell percentage (%)
100.0
90.0
80.0
70.0
60.0
50.0
40.0
30.0
20.0
10.0
0.0
0.01
0.1
1
10
Maprotiline Concentration (μM)

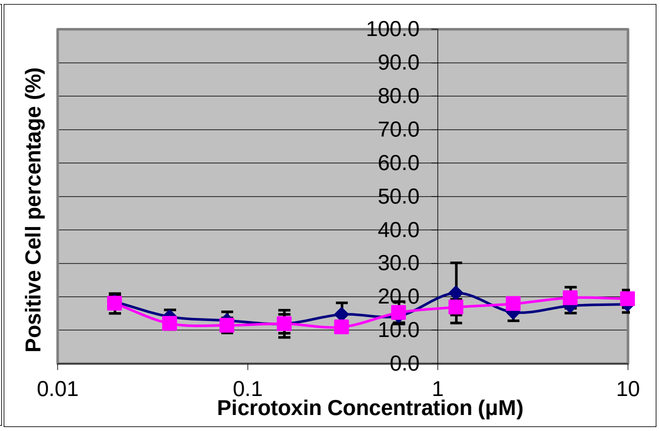
Positive Cell percentage (%)
100.0
90.0
80.0
70.0
60.0
50.0
40.0
30.0
20.0
10.0
0.0
0.01
0.1
1
10
Picrotoxin Concentration (μM)

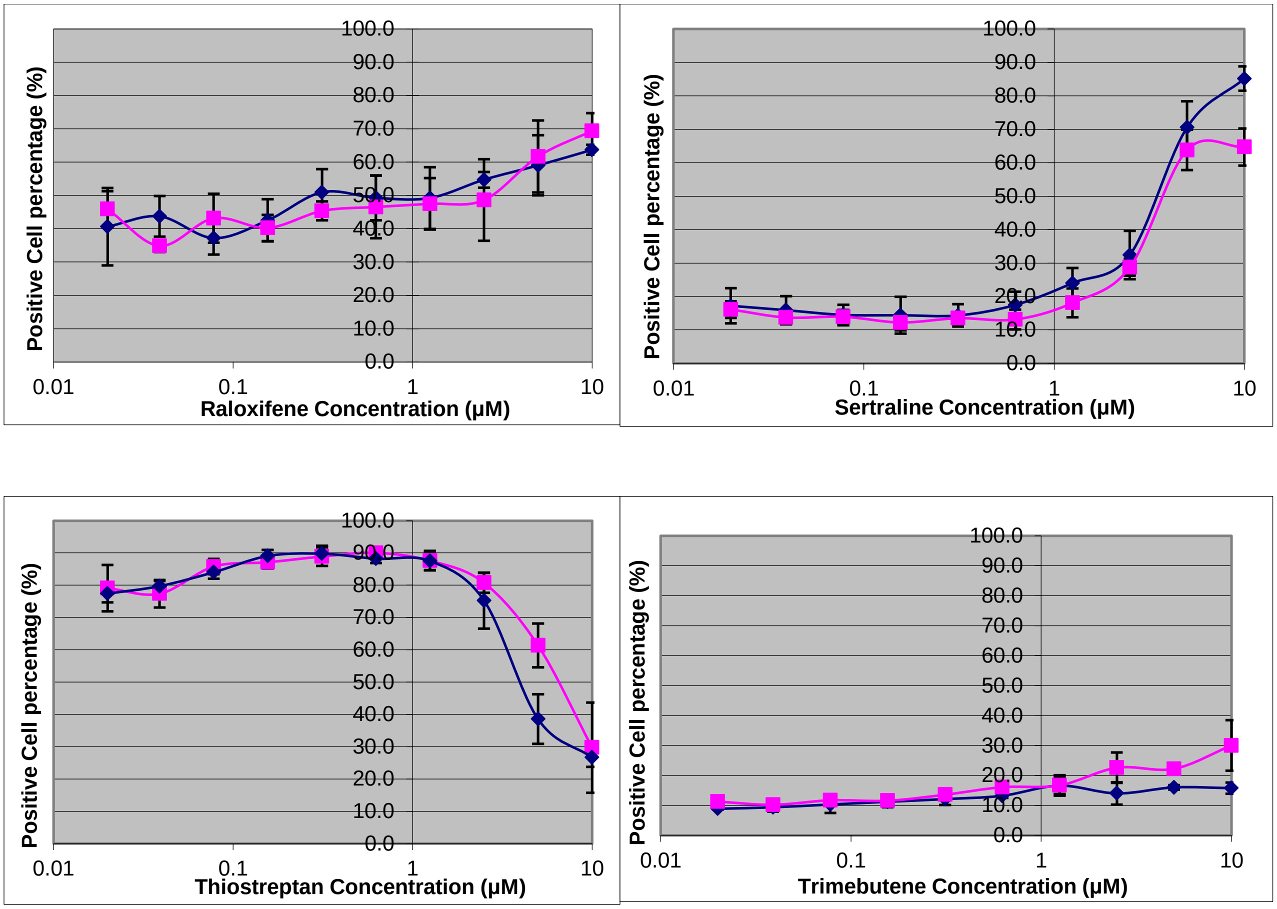

Figure S2. 16 drug dose response curves – comparisons between library and repurchased compound.

The horizontal axis is drug concentration (µM) in log scale. The vertical axis is the percentage of positive cells with LipidTOX fluorescent dye. We repeated the experiments twice for the repeatability. Blue, compound from the JHCCL; Red, repurchased (Sigma)

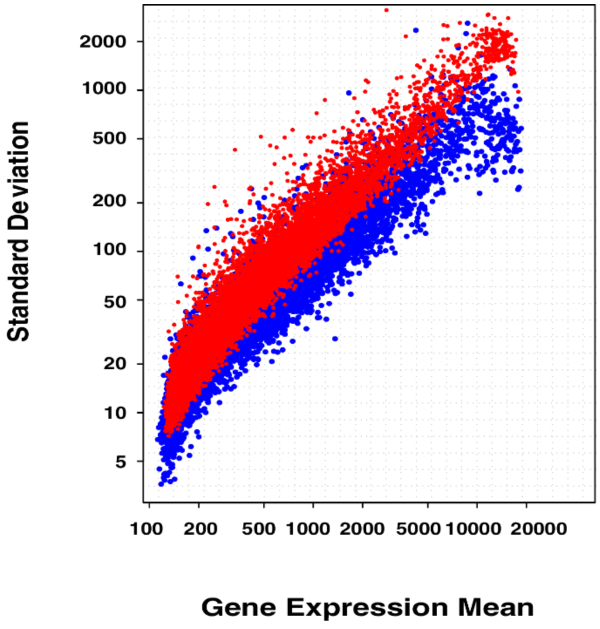

Figure S3. Error Model: MCF7 Cells Gene Expression levels vs. their standard deviations between untreated and drug-treated samples.

The horizontal axis represents the gene expression level measured by Illumina BeadChip while the vertical axis represents the corresponding standard deviation of gene expression levels across certain samples (both in log scale). The blue dots represent 14 samples of untreated MCF7 Cells while red ones represent 54 drug treated samples. The gene expressions vs. standard deviation curves of the drug-treated samples are significantly higher than those of untreated MCF7 cell samples.

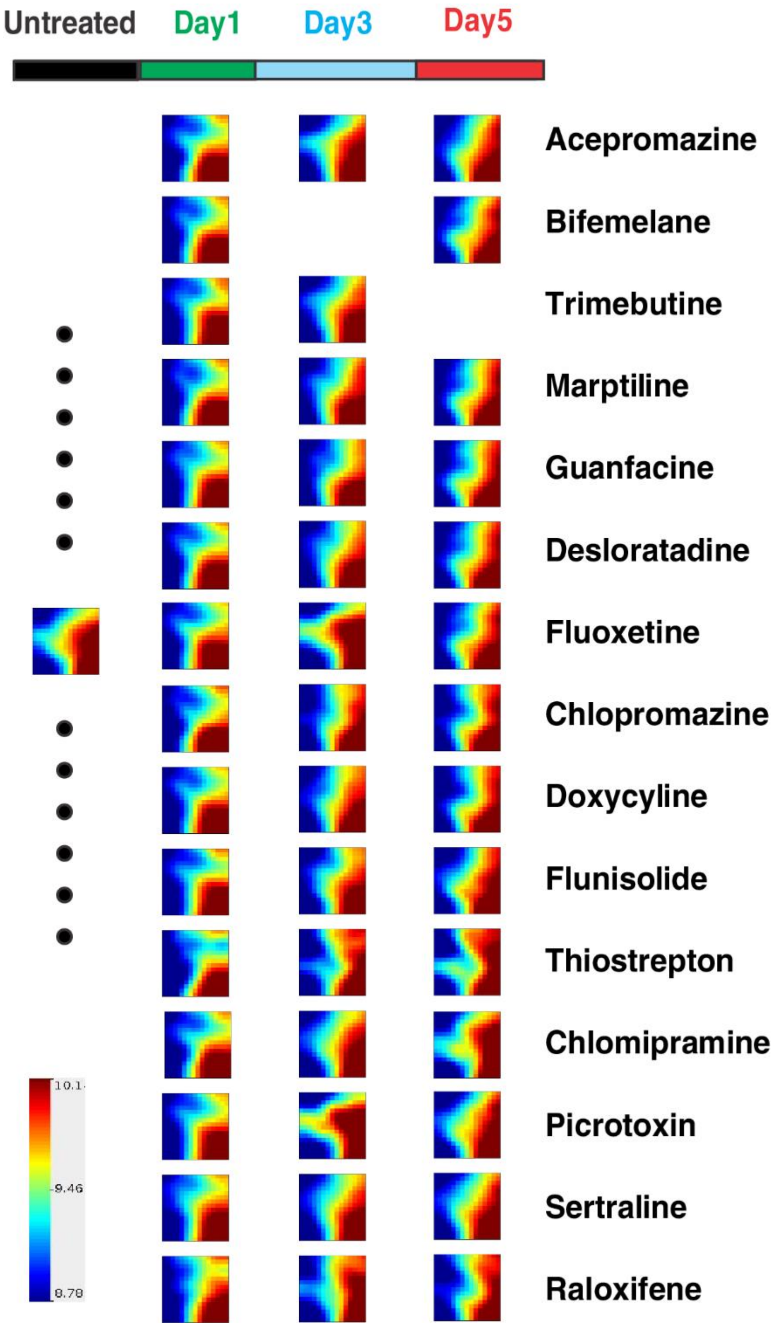

Figure S4. GEDI heatmap of untreated and 15 drug-treated MCF7 Cells Gene Expressions.

The GEDI heatmap project a high-dimensional transcriptome into a 2D self-organizing map to visualize the overall gene expression. Each pixel in the GEDI map (grid element) represents a mini-cluster of highly

similarly behaving genes. The pixel at the same position in each map represents the same genes. The color of each pixel represents the gene expression level. (Both *Befemelane* and *Trimebutine* have one transcriptome missed due to the failure to pass the quality control)

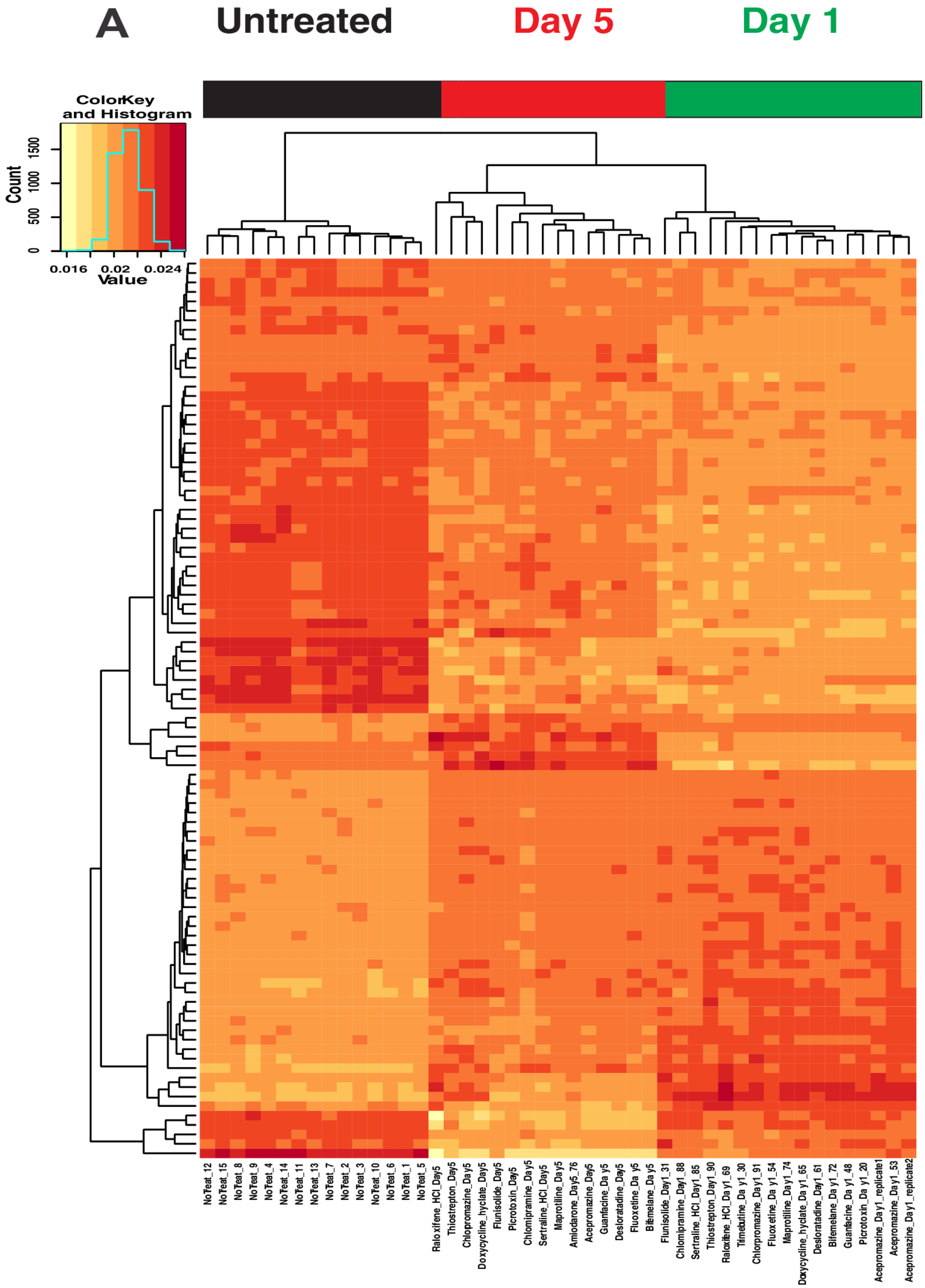
A
Untreated
Day 5
Day 1
Color Key
and Histogram
Count
0
500
1000
1500
0.016
0.02
0.024
Value
NoTeat_12
NoTeat_15
NoTeat_8
NoTeat_9
NoTeat_4
NoTeat_14
NoTeat_11
NoTeat_13
NoTeat_7
NoTeat_2
NoTeat_3
NoTeat_10
NoTeat_6
NoTeat_1
NoTeat_5
Raloxifene_HCl_Day5
Thiostrepton_Day5
Chlopromazine_Day5
Doxycycline_hyclate_Day5
Flunisolide_Day5
Picrotoxin_Day5
Chlomipramine_Day5
Sertraline_HCl_Day5
Maprotiline_Day5
Amiodarone_Day5_76
Acepromazine_Day5
Guanfacine_Day5
Desloratadine_Day5
Fluoxetine_Day5
Bifemelane_Day5
Flunisolide_Day1_31
Chlomipramine_Day1_88
Sertraline_HCl_Day1_85
Thiostrepton_Day1_90
Raloxifene_HCl_Day1_69
Trimebutine_Day1_30
Chlorpromazine_Day1_91
Fluoxetine_Day1_54
Maprotiline_Day1_74
Doxycycline_hyclate_Day1_65
Desloratadine_Day1_61
Bifemelane_Day1_72
Guanfacine_Day1_48
Picrotoxin_Day1_20
Acepromazine_Day1_replicate1
Acepromazine_Day1_53
Acepromazine_Day1_replicate2

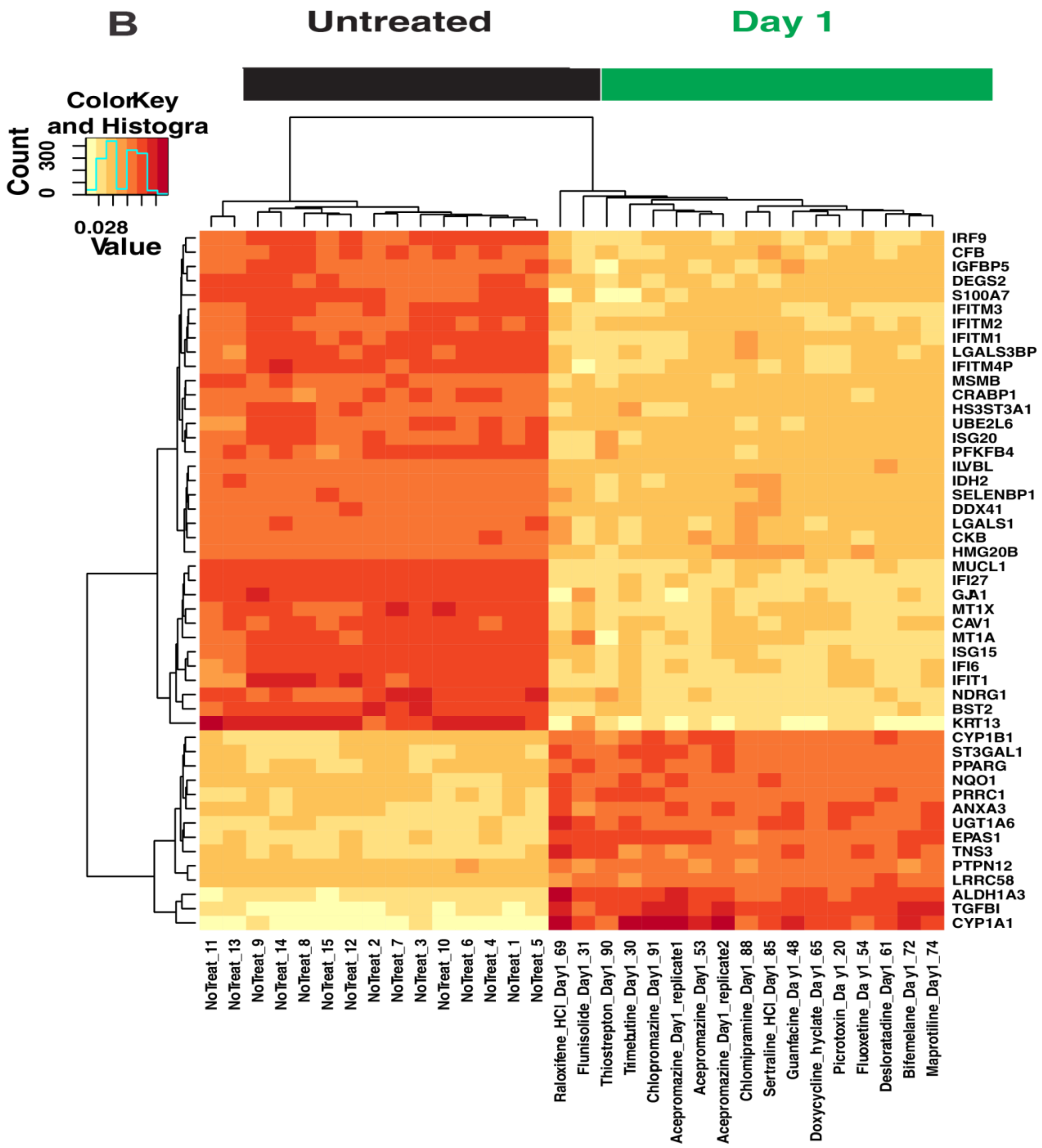

B
Untreated
Day 1
Color Key and Histogra
Count
0
300
0.028
Value
IRF9
CFB
IGFBP5
DEGS2
S100A7
IFITM3
IFITM2
IFITM1
LGALS3BP
IFITM4P
MSMB
CRABP1
HS3ST3A1
UBE2L6
ISG20
PFKFB4
ILVBL
IDH2
SELENBP1
DDX41
LGALS1
CKB
HMG20B
MUCL1
IFI27
GJA1
MT1X
CAV1
MT1A
ISG15
IFI6
IFIT1
NDRG1
BST2
KRT13
CYP1B1
ST3GAL1
PPARG
NQO1
PRRC1
ANXA3
UGT1A6
EPAS1
TNS3
PTPN12
LRRC58
ALDH1A3
TGFBI
CYP1A1
NoTreat_11
NoTreat_13
NoTreat_9
NoTreat_14
NoTreat_8
NoTreat_15
NoTreat_12
NoTreat_2
NoTreat_7
NoTreat_3
NoTreat_10
NoTreat_6
NoTreat_4
NoTreat_1
NoTreat_5
Raloxifene_HCl_Day1_69
Flunisolide_Day1_31
Thiostrepton_Day1_90
Trimebutine_Day1_30
Chlopromazine_Day1_91
Acepromazine_Day1_replicate1
Acepromazine_Day1_53
Acepromazine_Day1_replicate2
Chlomipramine_Day1_88
Sertraline_HCl_Day1_85
Guanfacine_Day1_48
Doxycycline_hyclate_Day1_65
Picrotoxin_Day1_20
Fluoxetine_Day1_54
Desloratadine_Day1_61
Bifemelane_Day1_72
Maprotiline_Day1_74

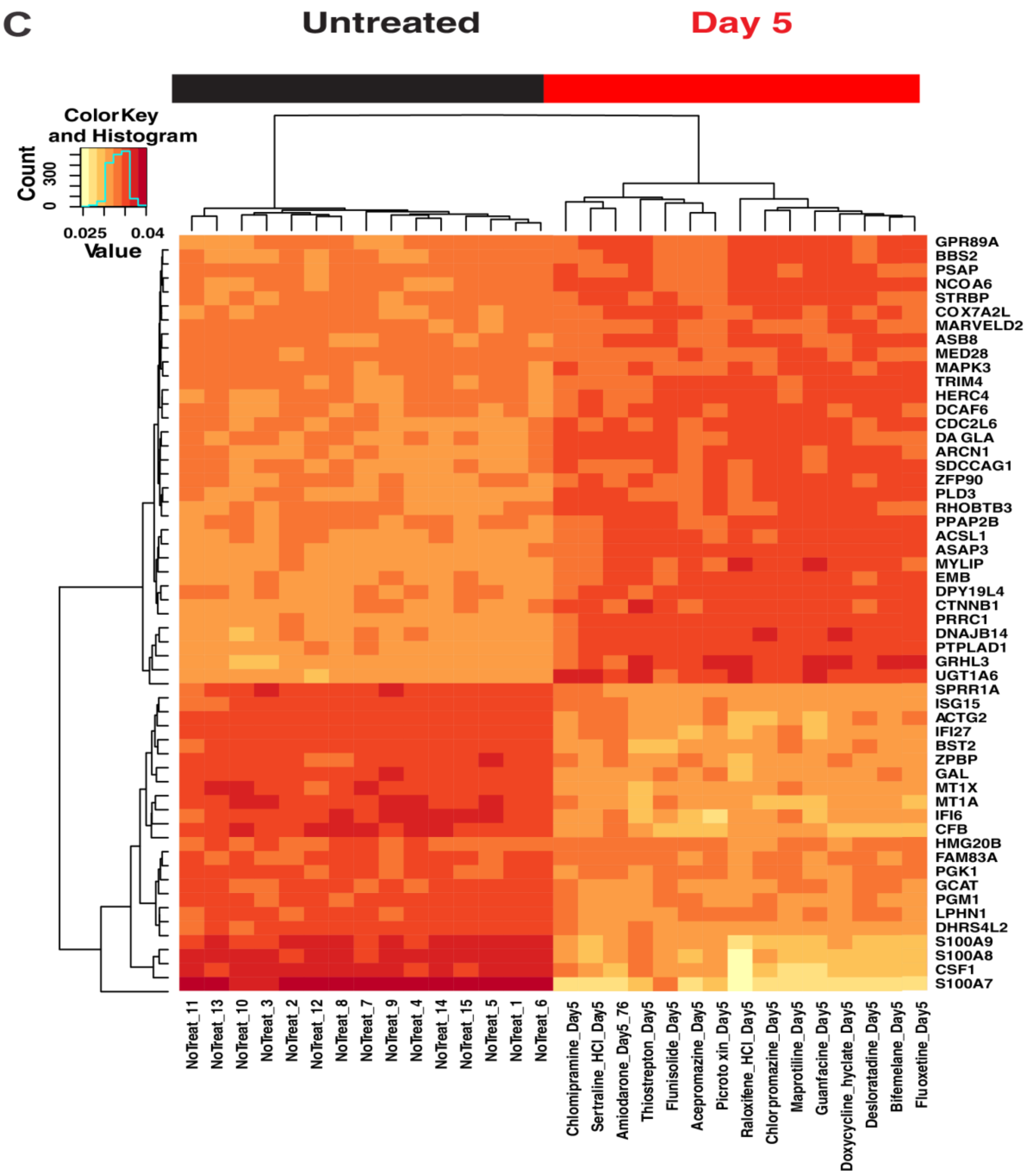

Figure S5. Heatmap of the differentially expressed genes identified using SAM.

(**A**) The heatmap of the differentially expressed genes between the samples untreated (at Day 0), drug treated samples at Day 1 and Day 5 based on SAM multiclass analysis; (**B**) The differentially expressed genes between the samples at Day 0 and Day 1 based on SAM analysis; (**C**) The differentially expressed genes between the samples at Day 0 and Day 5 based on SAM analysis.

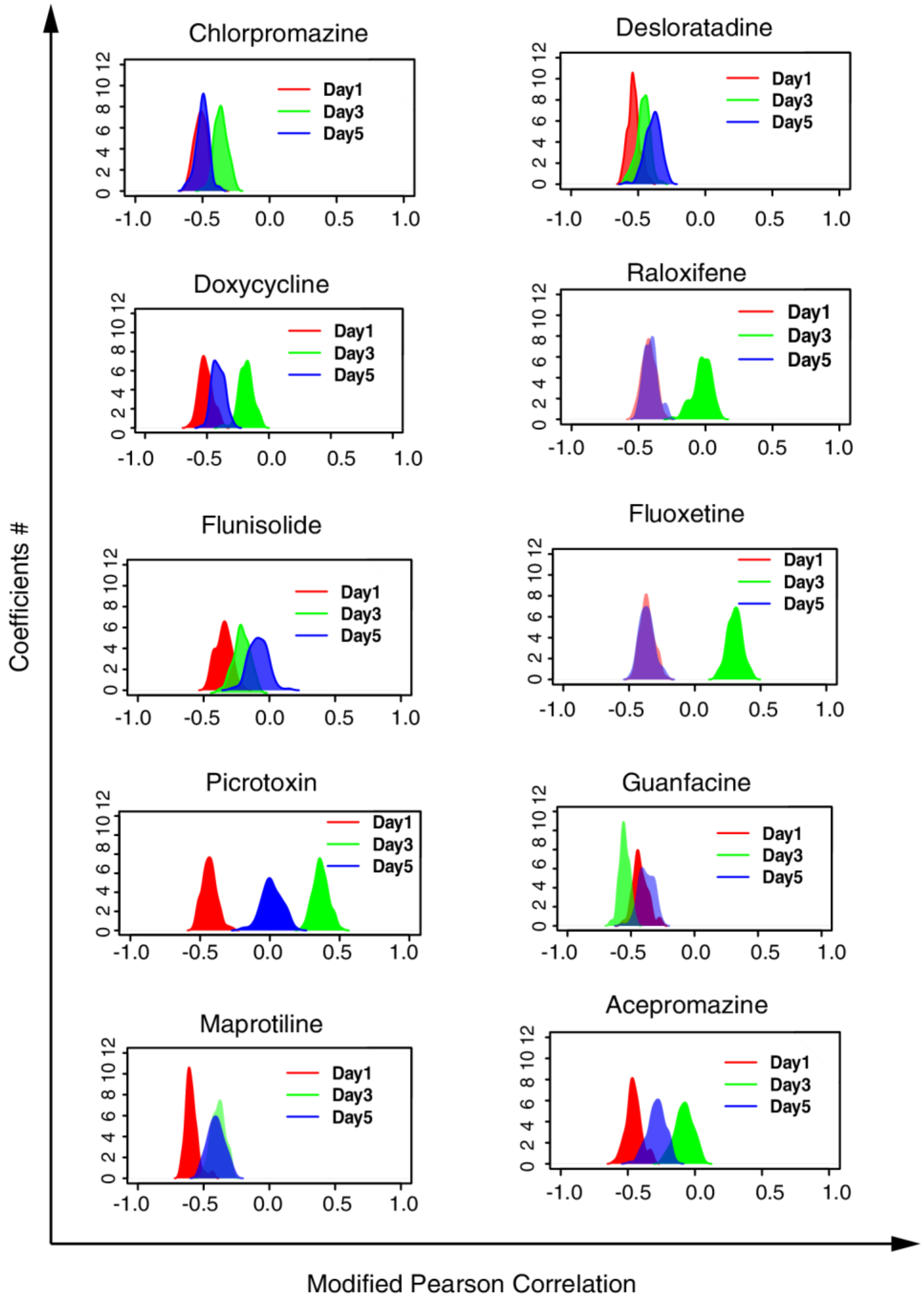

Figure S6. The modified Pearson correlation coefficients distribution of MCF7 cell samples between Day 0 and Day 1,3,5

From 100-randomly chosen sets of 500 genes out of the entire transcripts of each drug at different time points Indicated by colors), we calculated the distribution of the modified Pearson correlation coefficients

between the drug-treated and the untreated samples (see details in [1]). Except for Desloratadine, Flunisolide, Guanfacine and Maprotiline, the gene expressions of the drug-treated samples are less correlated between Day 0 and Day 3 than the ones between Day 0 and Day 1 / Day 5. The gene expressions of the drug-treated samples were diverging and converging during the transition.

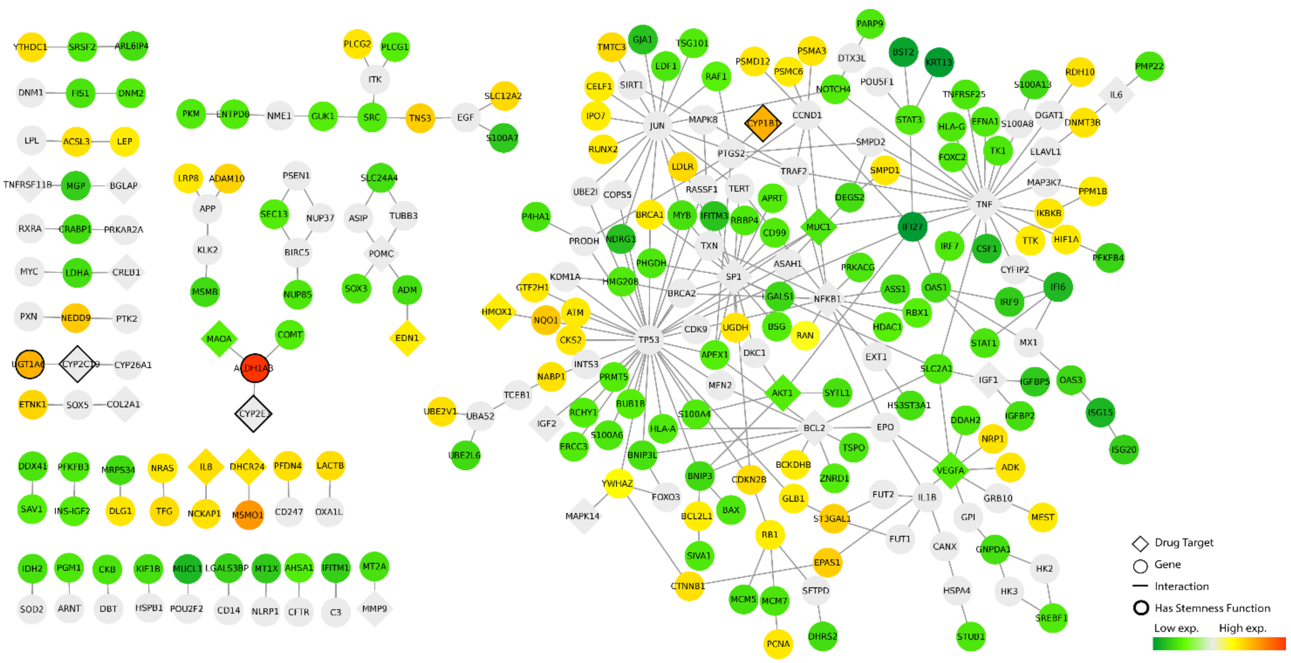

Figure S7. The CAP-Net analysis of the transcriptome of drug-treated sample at Day 1

CAP-Net shows the downstream commonly affected pathways after 1 day drug treatments. Diamonds and circles respectively represent targets and differentially expressed genes. The color of a node shows the average expression level of the corresponding gene. If a gene has a stemness function, it is marked by bold black line.

Supplemental Tables

Table S1-1. List of 16 drugs names, optimal dose, cell differentiation efficiencies

No.	Full Name	Indication	C (μM)	p (%)	Survived Cells (%)
1	Acepromazine	Sedative	10.00	45.2	73.3
2	Amiodarone	Antiarrhythmic (class III)	5.00	44.8	48.3
3	Bifemelane	Nootropic	5.00	45.2	70.1
4	Chlorpromazine	Antiemetic	5.00	46.3	70.7
5	Clomipramine	Antidepressant	5.00	33.7	83.6
6	Desloratadine	Antihistaminic	10.00	55.5	65.3
7	Doxycycline	Antibiotic	5.00	38.3	86.0
8	Fluoxetine	Antidepressant	10.00	47.9	70.3
9	Flunisolide	Glucocorticoid	10.00	29.1	99.6
10	Guanfacine	Antihypertensive	10.00	46.3	59.6
11	Maprotiline	Antidepressant	5.00	35.8	85.7
12	Picrotoxin	Nootropic	10.00	19.7	100.0
13	Raloxifene	Bone resorption inhibitor	0.16	41.4	100.0
14	Sertraline	Antidepressant	10.00	67.2	66.7
15	Thiostrepton	Antibiotic	0.08	84.9	100.0
16	Trimebutine	Antispasmodic	10.00	22.9	75.4

Table S1-2. Drug concentrations for transcript profiling

No.	Drug name	Concentration (μM)
1	Acepromazine	10
2	Amiodarone	5
3	Bifemelane	5
4	Chlomipramine	5
5	Chlorpromazine	10
6	Desloratadine	10
7	Doxycycline hyclate	10
8	Fluoxetine	10
9	Flunisolide	10
10	Guanfacine	10
11	Maprotiline	10
12	Picrotoxin	10
13	Raloxifene HCl	5
14	Sertraline HCl	5
15	Thiostrepton	1
16	Trimebutine	10

Table S2. Differentially expressed genes identified by SAM analysis

Day 0 vs Day 1 vs Day 5 (138	Day 0 vs Day 1 (49 genes)	Day 0 vs Day 5 (41 genes)
ABCC3	ALDH1A3	ACSL1
ACSL1	ANXA3	ACTG2
ALDH1A3	BST2	ARCN1
ANKRD13C	CAV1	ASAP3
ARCN1	CFB	BBS2
ARL6IP4	CKB	BST2
ARMCX6	CRABP1	CDC2L6
ASAP3	CYP1A1	CFB
ASB8	CYP1B1	COX7A2L
ATAD1	DDX41	CTNNB1
BNIP3	DEGS2	DAGLA
BRF1	EPAS1	DCAF6
BST2	GJA1	DHRS4L2
C10ORF116	HMG20B	DPY19L4
C17ORF90	HS3ST3A1	GAL
CAV1	IDH2	GCAT
CFB	IFI27	GRHL3
CKB	IFI6	HMG20B
CNN2	IFIT1	IFI27
COX7A2L	IFITM1	MED28
CRABP2	IFITM2	MT1A
CSF1	IFITM3	MT1X
CTNNB1	IFITM4P	PGK1
CXORF39	IGFBP5	PGM1
CYP1B1	ILVBL	PLD3
DAGLA	IRF9	PRRC1
DDR1	ISG15	PTPLAD1
DDT	ISG20	RHOBTB3
DDX41	KRT13	S100A7
DEGS2	LGALS1	S100A8
DNAJB14	LGALS3BP	S100A9
DPY19L4	LRRC58	SDCCAG1
DYNC1LI1	MSMB	SPRR1A
EHD1	MT1A	UGT1A6
EMD	MT1X	ZFP90
ENO1	MUCL1	
EPAS1	NDRG1	
FBXL18	NQO1	
FIS1	PFKFB4	
FLVCR2	PPARG	
FOXJ3	PRRC1	
GAL	PTPN12	
GAPVD1	S100A7	
GCAT	SELENBP1	
GPER	ST3GAL1	
GPI	TGFBI	
GPX2	TNS3	
GRHL3	UBE2L6	
HCFC1R1	UGT1A6	
HERC4	UGT1A6	
HIP2		
HLA-DRB5		
HLA-H		
HMG20B		
IDH2		
IFI27		
IFI6		
IFIT1		
IFITM1		

IFITM2
IFITM3
IFITM4P
IGFBP2
ILVBL
IRF9
ISG15
KLK11
LCN2
LGALS3BP
LIN7C
LMTK3
LOC728037
LPHN1
LRRC58
MAFB
MAPK3
MCOLN2
ME1
MEST
MGAT2
MRPL54
MT1A
MT1X
MTE
MUCL1
MYLIP
NCOA6
NDRG1
NFE2L2
OAS1
OBFC2A
P2RX2
PANX2
PARP12
PAWR
PCP4
PFDN4
PFKFB4
PGK1
PGM1
PHACTR2
PIH1D1
PLD3
PPARG
PPM1B
PREPL
PRRC1
RHOBTB3
RSRC1
S100A7
S100A8
S100A9
SELENBP1
SERPINA1
SEZ6L2
SIKE
SLC2A1
SP110
SPRR1A
SQLE
STK3
STRBP
SULF2

SYTL1
TGFBI
TMEM1
TMEM43
TMEM72
TMEM79
TNNT1
UBE2L6
UGT1A6
VPS35
WDR54
YIPF4
YTHDC1
ZFP90
ZNF721

Table S3. The gene lists of four different dynamic patterns (C1 – C4)

C1 (Const, diverge-converge)	C2 (Const, Concordant)	C3(change, diverge-converge)	C4 (change, concordant)
ACOT11	ABCA12	ACSL1	7A5
ACOX2	ABCC3	ACSL3	AAGAB
ACSS2	ABHD3	AHCTF1	AASDHPPT
ANKRD11	ACAP2	AHNAK	ACO1
ASPM	ACAT2	AP1M1	ACP1
BRCA2	ACLY	ARHGEF16	ACTG2
C1ORF52	ACOT4	ARL6IP4	ACTR3
COL4A5	ACTR1B	ASAP3	ACVR1
COMMD7	ACTR6	ASB8	ADAM10
COX7B	ADCY1	ATP5B	ADNP
CP110	ADM	ATP9A	AES
CRIP2	AFAP1L2	BANF1	AGTPBP1
CUL5	AGL	BBS2	AHSA1
CUTA	AGR2	BCL6	AIF1L
ELF3	AHSA2	BTG1	AKAP9
ETFA	AIDA	BUB3	AKIRIN2
FAM96A	AIP	C15ORF21	ALDH4A1
FDFT1	AK3L1	C18ORF25	ALDH5A1
FTHL3	AKAP11	C19ORF33	ALS2
GDPD3	AKR7A2	C2ORF64	ANAPC5
GSDM1	AKR7A3	C3ORF59	ANKHD1
H3F3A	ALDOC	CCDC124	ANKIB1
HADH	ALG1L	CCDC23	ANKRD13C
HDAC1	ALKBH2	CCDC72	ANKRD47
HIST1H2BE	AMMECR1	CDC2L6	ANKRD6
HMOX1	ANLN	CFL1	APRT
IDH2	ANP32B	CHCHD4	ARCN1
IFT74	AP1G2	CHMP2B	ARHGAP12
IMPA1	AP1S3	CKS1B	ARHGAP18
KBTBD8	AP2S1	CRY2	ARHGEF19
KDM5B	APH1A	CSNK2B	ARHGEF5L
KIAA1033	ARF1	CSTB	ARID5B
KLHDC5	ARF5	DAPP1	ARL8B
KRT8	ARFGEF2	DBNDD1	ARMCX6
KRT80	ARHGAP21	DDX41	ASF1B
LAD1	ARID1A	DHCR7	ATAD1
LOC653604	ARL2	DUSP3	ATF1
LOC678655	ARL6IP5	DYRK2	ATP5D
MAP7	ARPC5	EIF3M	ATP5G1
MGC16703	ARRDC4	EWSR1	ATP6AP2
MGST2	ASCL2	FAM117B	ATP6V0E2
MIF	ASH1L	FAM129B	ATP6V1G1
MRFAP1	ASS1	FAM179B	AUTS2
NDUFA1	ATG16L1	FAM43A	B3GAT3
NUBPL	ATIC	FAM96B	B4GALT1
OCIAD2	ATOX1	FLJ38482	BAG3
PAFAH1B3	ATP2B1	FLJ44124	BAMBI
PMEPA1	ATP2C1	FTHL11	BAZ2B
POMP	ATP6V0C	GAK	BCAR3
PRDX1	ATP6V1B1	GCA	BCL2L1
RCC2	ATP6V1F	GFM1	BEND6
RHBDD2	ATPIF1	GLTP	BICD2
RHEB	ATR	HIST1H4E	BIVM
RNASET2	B4GALNT1	HMCN1	BMI1
RPL11	BAIAP2L1	HMGCS1	BNIP3
RPL15	BAT1	HSPA1A	BNIP3L
RPS3A	BAT5	ID2	BRF1
RPS6KB1	BAZ1A	IDI1	BRF2

S1PR3	BBS7	KIAA0430	BRPF3
SEMA3E	BBX	KLF9	BRSK1
SH3GLB2	BCAP29	KLHL12	BRWD3
SHCBP1	BCKDK	LMTK3	BSCL2
SIAH2	BCL2L2	LSM7	BST2
SLC16A12	BCL7C	MCM2	BTF3L4
SLC36A4	BIRC5	MED28	C12ORF24
SLTM	BMS1	MED30	C12ORF41
SQSTM1	BNIPL	METAP2	C13ORF23
TBC1D8	BRD3	MRPL46	C13ORF37
TINP1	BSG	MRPL51	C14ORF4
TMEM165	BUB1	MT1A	C15ORF29
TMEM205	C10ORF118	MTX2	C16ORF13
TMEM9	C10ORF119	MYLIP	C16ORF58
TMSB10	C10ORF46	NARS	C17ORF58
TTYH3	C10ORF58	NAT12	C1ORF218
UBR3	C10ORF59	NCOA6	C1ORF63
VN1R2	C11ORF10	NDUFA13	C1ORF71
VPS28	C11ORF60	NFIB	C1QTNF6
VPS72	C11ORF61	NMD3	C21ORF58
WDR67	C12ORF10	NUDT7	C2ORF30
XPNPEP1	C12ORF35	PABPC1	C2ORF54
XPNPEP3	C12ORF44	PDLIM1	C2ORF55
ZBTB42	C12ORF57	PFDN4	C3ORF57
ZNF146	C14ORF126	PPCS	C4ORF23
	C14ORF32	PPP1CC	C6ORF125
	C16ORF35	PRRG1	C7ORF28B
	C16ORF75	PSMA5	C7ORF42
	C16ORF91	PTPLB	C9ORF127
	C17ORF61	RAB10	C9ORF142
	C17ORF90	RAB11A	CAMSAP1L1
	C17ORF98	RAB5B	CAND1
	C19ORF22	RBM47	CAPNS1
	C19ORF30	RBPMS2	CAPZA1
	C19ORF43	REPIN1	CBR3
	C1ORF123	RFX7	CBS
	C1ORF128	RGPD2	CBX2
	C1ORF55	RGS17	CBX6
	C1ORF96	RHBDF1	CCBE1
	C20ORF199	RHOBTB3	CCDC128
	C20ORF52	RNF114	CCDC132
	C3ORF75	RNF181	CCDC22
	C5ORF33	RNF216	CCDC50
	C7ORF20	ROCK1	CCDC83
	C7ORF30	RPL26	CCDC85A
	C7ORF43	RPL26L1	CCDC85B
	C7ORF50	RPL27	CCDC90A
	C7ORF68	RPL7L1	CCDC90B
	C9ORF102	RPS7	CCDC91
	C9ORF169	SAFB	CCDC93
	C9ORF41	SEMA3B	CCM2
	CAB39	3-Sep	CD164
	CAB39L	SLC25A16	CD46
	CABYR	SLC38A6	CD96
	CAMK2B	SNORD3D	CDC45L
	CAMTA1	SNX27	CDCA7
	CAPG	SPAST	CDKN2B
	CAPN13	SPRYD5	CELSR2
	CAPN7	SRM	CENPO
	CARHSP1	STAG3	CEP27
	CASC4	STARD10	CETN2
	CASC5	STAT6	CFB
	CAT	STC1	CFL2
	CBX3	STRA13	CGGBP1

CCDC112	SUCLG2	CHES1
CCDC120	SULT1A1	CHMP4C
CCDC18	SUMF1	CHP
CCDC41	SYPL1	CHST1
CCDC56	TACSTD1	CIDEC
CCNC	TBL3	CLDN3
CCNE2	TCEA3	CLK4
CCNJ	TMEM126B	CMBL
CCNYL1	TMEM66	CMTM8
CD63	TMEM9B	CNN2
CD97	TOMM70A	COBLL1
CD99	TRMT5	COMT
CDC2	TUBB2C	COMTD1
CDC73	UBC	COX10
CDH3	UBLCP1	COX6B1
CDK4	USP38	CPD
CDKN1A	YBX1	CPEB4
CDR2L	ZC3H3	CPOX
CDS1	ZNF281	CRELD2
CEBPZ		CRIPT
CENPA		CTNNB1
CEP55		CTNNBL1
CHCHD5		CTNND2
CHKA		CWF19L1
CHML		CXORF26
CHORDC1		CXORF39
CHPT1		CYP1B1
CICK0721Q.1		DAGLA
CIRBP		DAZAP2
CKB		DBN1
CKMT1A		DBT
CKMT1B		DCAF6
CKS2		DCTN4
CLCN3		DCUN1D1
CLDN7		DDEF2
CLINT1		DDT
CLIP1		DDX17
CLIP4		DDX6
CLK2		DEGS2
COG4		DHRS4
COMMD4		DHRS4L2
COPS6		DHRS7
COX11P		DIRC2
COX5B		DKC1
CRABP2		DLG1
CRISPLD2		DMKN
CRYZ		DNAJB14
CSNK1G3		DNAJC9
CSRP1		DNAL4
CXXC5		DNLZ
CYC1		DPM3
CYHR1		DPY19L4
DAD1		DR1
DARS		DRAM1
DBNL		DYNC1LI1
DCI		E2F2
DCP2		E2F6
DCTN1		EDF1
DCTN2		EGLN3
DCTPP1		EHD1
DDAH2		EHD4
DDB2		EIF2AK3
DDHD1		EIF3C
DDR1		EIF3D

DDX24
DGUOK
DHCR24
DHFRL1
DHRS3
DHX32
DHX40
DMXL1
DNAJB4
DNMT3B
DNPEP
DOCK6
DOK4
DOK7
DPP7
DSC2
DSTN
DUSP4
DYNLRB1
ECH1
ECHDC2
EDC3
EDEM3
EEF1D
EEF1E1
EEF1G
EFCAB2
EFHA1
EFHD1
EFNA1
EGFL7
EIF3A
EIF3CL
EIF3G
EIF4E2
EIF4E3
EIF4G2
ELK1
ELOVL5
ELOVL7
EML1
ENTPD8
EPB41L5
EPHB4
ERCC1
ERGIC2
ERGIC3
ERI3
ESCO2
ETNK1
EXOC3
EXOSC7
FAM104A
FAM108B1
FAM116A
FAM161A
FAM38A
FAM57A
FAM65A
FAM69A
FAM72A
FAM80A
FAM84B
FAM91A1

ELAVL3
ELF5
ELK4
ELOF1
EMB
EMD
ENO1
ENO2
ENPP4
ENSA
EPCAM
ERICH1
ERMP1
ESF1
EXD2
EXO1
EXOC4
EXOC8
EXOSC5
FAHD2A
FAM105B
FAM107B
FAM120A
FAM120B
FAM160B1
FAM188A
FAM45A
FAM62B
FAM83A
FAM98A
FAU
FBL
FBXL18
FBXO18
FBXO33
FGD3
FGF13
FGFR1OP
FIS1
FLAD1
FLNB
FLVCR2
FNBP1L
FOXC2
FOXJ3
FRAT2
FSCN1
FTSJ3
G3BP1
GAB1
GAL
GALK2
GAPVD1
GATS
GCAT
GEMIN4
GK5
GKAP1
GLB1
GLS
GNGT1
GNPDA1
GOLT1B
GPI

FAT1
FBXL15
FBXL17
FBXO30
FBXO32
FIBP
FKBP9L
FLJ33630
FLOT1
FLRT3
FNBP1
FTHL12
FTSJD1
GABRB3
GAN
GAPDH
GCH1
GCLC
GCLM
GCNT1
GDI2
GFPT1
GNA13
GNAI3
GNG12
GON4L
GPER
GPKOW
GPR65
GPX2
GPX3
GRAMD3
GRK6
GSN
GSTO2
GTF2F2
GTF2I
GUF1
H2AFV
H2AFY2
HACL1
HAGH
HCFC1R1
HCFC2
HDGF
HDLBP
HES4
HHEX
HIATL1
HIST1H2BC
HIST1H2BH
HIST2H2AC
HIST3H2A
HLA-A
HLA-DRB1
HLA-DRB5
HLA-DRB6
HLA-G
HLA-H
HMGCR
HNRNPA1L2
HNRNPH3
HNRNPK
HNRPH3

GPR137B
GPR89A
GRAMD1A
GRHL3
GRN
GSDMC
GSTO1
GTF2A2
GTF3C4
GTF3C5
GTSE1
GUK1
H19
HARS2
HBP1
HDHD3
HEBP1
HERC4
HINT2
HIP2
HIST1H2AB
HIST1H3E
HIST1H4H
HIST1H4J
HIST3H3
HMG20B
HMGN4
HNRNPD
HNRNPM
HOXC6
HRASLS3
HSBP1
HSPC111
HTR3A
IFI6
IFIT1
IFITM1
IFITM3
IFITM4P
IGFBP4
IGSF5
ILF2
ILVBL
INS-IGF2
IRF9
IRS1
ISCU
ISG15
ISG20
JMJD1C
JMY
KAT2B
KCTD7
KIAA0101
KIAA0528
KIAA1267
KIAA1598
KIDINS220
KIF1A
KIF22
KLC1
KLHDC10
KLHDC8B
KLHL2

HOXA10
HOXA6
HOXC13
HRSP12
HSD17B10
HSP90AA1
HSPBL2
HSPD1
HTATIP2
HTRA1
IBTK
ICAM3
IDS
IFFO2
IFI27L1
IFI35
IFITM2
IFNGR1
IGFBP2
INSIG1
INSM1
INTS3
INTS8
IPO7
ITGAV
ITGB4
ITPRIP
ITSN1
JAKMIP3
KCNH6
KCTD9
KGFLP1
KHDRBS1
KIAA1128
KIAA1147
KIAA1324
KIAA1524
KIAA1688
KIAA1737
KIAA1804
KIF14
KIF3A
KLHL20
KLHL7
KLHL9
KPNA4
KREMEN2
KRT16
KRT19
KRT20
KRT81
KRTCAP2
KTN1
LACTB
LAGE3
LAMB2
LCMT1
LCOR
LEPROTL1
LNX2
LOC100128731
LOC399744
LOC401233
LOC550643

KPNA3
KRT10
KRT17
LAIR1
LAMB1
LARGE
LCN2
LDHA
LEPROT
LGALS3BP
LIMK2
LIN7A
LIN7C
LMCD1
LMNA
LMO4
LOC100129055
LOC100132247
LOC100132707
LOC100134868
LOC389286
LOC389834
LOC646996
LOC728037
LOXL1
LPHN1
LRCH4
LRRC1
LRRC16B
LRRC42
LRRC58
LRRCC1
LSM4
LTA4H
LTB
MAB21L2
MAL2
MAN2A1
MAN2B2
MAP2K1
MAP2K1IP1
MAP3K5
MAP4K2
MAPK3
MAR6
MARVELD2
MAT2A
MBD6
MBNL2
MCM3
MCM4
MCM5
MCOLN2
MEA1
MED20
MEST
METTL11A
METTL7A
MEX3C
MFAP2
MFSD10
MGAT2
MGAT4B
MGC10997

LOC552889
LOC606724
LOC653994
LOC90624
LPIN1
LRP5
LRP8
LRPAP1
LRRC8B
LSM1
LYAR
LYN
LYPD6
M6PRBP1
MACROD1
MAD2L2
MAN1B1
MANBAL
MAP9
MAPRE3
MAR3
MAST2
MAT2B
MBNL1
MBNL3
MBOAT7
MC1R
MCF2L2
ME1
MEIS3
MEIS3P1
METRN
MFF
MFN1
MFSD6
MGC12965
MKI67IP
MLST8
MMD
MNAT1
MPHOSPH10
MPI
MPP6
MPV17
MRLC2
MRPL1
MRPL22
MRPL23
MRPL34
MRPL43
MRPS12
MT1F
MTERFD1
MTFMT
MTMR11
MTMR9
MTSS1
MUC1
MXD3
MYL6B
MYO18A
NAB2
NACA2
NAP1L2

MID2
MINPP1
MIR1185-1
MITD1
MNX1
MORC3
MPG
MRGPRF
MRPL12
MRPL2
MRPL4
MRPL42P5
MRPL54
MRPS15
MRPS23
MRTO4
MSX2
MT1G
MT1X
MT2A
MTE
MTUS1
MXD1
MYCBP2
MYH10
MYL12A
MYO1B
MYO5A
MYO6
MYPOP
MZF1
NBEA
NCAM2
NCOA1
NDRG1
NDUFA4
NEDD1
NEK8
NELL2
NEURL4
NFE2L2
NMB
NME1
NNT
NOL10
NOL3
NPNT
NR1D1
NR3C1
NRBF2
NRCAM
NSDHL
NT5E
NUBP2
NUCB1
NUCB2
NUP85
OAS1
OMA1
OR8B12
OSBPL8
OSGIN2
P4HA2
PA2G4

NARF
NCAPH
NCKAP1
NDEL1
NDFIP1
NDFIP2
NDUFA11
NDUFA2
NDUFAF3
NDUFB11
NDUFS5
NETO1
NHP2
NIT1
NKX3-1
NLGN2
NME4
NMRAL1
NR2C2
NR2F2
NRAS
NSMCE1
NT5C
NT5C2
NT5DC3
NTN4
NUMA1
OAS3
OBFC2A
OGFR
OIP5
OKL38
OPA1
ORC2L
OTUB2
OTUD6B
PALLD
PAM
PANX2
PAPOLA
PAQR3
PARK7
PARP10
PARP4
PARP9
PBX2
PCDHB2
PCGF5
PCNA
PCP4
PDSS2
PFKL
PFN2
PGA3
PGLS
PHAX
PHB2
PHC3
PHF11
PHF20L1
PHOSPHO2
PHPT1
PHTF2
PI4KB

PAPD4
PAPSS2
PARP12
PAWR
PBLD
PBX3
PCSK6
PDCD2L
PDLIM7
PDPR
PDS5A
PDZRN3
PERP
PFKFB3
PFKFB4
PGAM1
PGK1
PGM1
PHACTR2
PHACTR4
PHB
PHYH
PIGK
PIH1D1
PIK3CA
PKD2
PKMYT1
PKP4
PLCG2
PLD3
PLEC1
PLS1
POLD4
POLR1D
PPAP2B
PPIL2
PPM1B
PPM2C
PPP2R3A
PPP2R4
PPP2R5D
PRELID1
PREPL
PRICKLE2
PRICKLE4
PRKCSH
PRR15L
PRR7
PRRC1
PSMB1
PSMB10
PSMC3
PSMD3
PSMF1
PTDSS1
PTGER4
PTPLAD1
PTPN3
PTPN4
PTPRF
PTPRG
PTTG1IP
PUF60
PURB

PIAS3
PICALM
PIGC
PKM2
PKP1
PLOD3
POLR2H
POLR2I
PP8961
PPAP2A
PPARG
PPIAL4A
PPIF
PPM1E
PPP1CA
PPP2R1A
PPP4C
PPP4R1
PPP4R2
PQBP1
PRDM4
PRDX5
PRIM2
PRKRIR
PRPF19
PRPF31
PRR14
PSENEN
PSMA3
PSMC2
PSME1
PSMG4
PTGR1
PTMA
PTPN11
PURA
PYGB
PYGL
RAB25
RAB27B
RAB32
RAB33B
RAB3GAP2
RAB3IP
RAB5C
RAB8A
RABAC1
RABEP2
RABGAP1
RAF1
RALBP1
RALGDS
RAMP1
RAP1GDS1
RAP2C
RAPGEFL1
RBM12B
RBM42
RBX1
RDH10
RDH11
REEP3
RFK
RMND5B

PUSL1
PYCRL
QTRT1
R3HDM2
RAB22A
RAB2A
RAD21
RALB
RAP1GAP
RAPGEF2
RASA1
RB1CC1
RBL2
RBM45
RDH14
RECQL4
RELB
REXO2
RFX3
RHBDF2
RHOQ
RHOU
RNASEL
RNF11
RNF34
RNF40
RNU4ATAC
RNU5A
RP11-529I10.4
RPL36A
RPL39L
RPP21
RPS21
RPS4Y2
RRM2
RRP1
RSRC1
RUNDC3B
RXRA
S100A12
S100A4
S100A7
S100A8
SALL4
SAMD9
SASH1
SCYL2
SDCCAG1
SDF2L1
SDHB
SEC24B
SENP6
SENP7
SEPT11
SERPINA1
SERPINB6
SF3A2
SF3A3
SFN
SFRS12
SFRS2
SFRS2B
SFRS2IP
SFRS6

RNASEH2A
RNASEK
RNF19B
RNU4-1
ROBLD3
ROMO1
RPE
RPL13A
RPL17
RPL3
RPL36
RPN2
RPRD1A
RPS15
RPS24
RPS4X
RPS6KB2
RPS9
RPSA
RPUSD3
RSU1
RTCD1
RTKN
RUFY2
RUNX2
S100A14
S100A16
SACS
SALL2
SAPS1
SBF1
SBNO1
SCP2
SCPEP1
SDCBP
SDF2
SDF4
SEC23B
SEC23IP
SEC24A
SEC24D
SEMA4B
SEMA6B
SERTAD1
SEZ6L2
SFRS14
SFXN5
SH3D19
SHISA5
SIGIRR
SIK3
SIN3A
SIPA1
SIX5
SLC16A6
SLC22A4
SLC25A46
SLC29A3
SLC2A1
SLC2A6
SLC30A6
SLC31A2
SLC35A1
SLC35A3

SGSH
SGSM2
SGTA
SH3BP1
SH3YL1
SHRM
SIKE
SKP1
SLAIN1
SLC25A1
SLC25A12
SLC27A3
SLC35F5
SLC38A2
SLC44A1
SLC44A2
SLC48A1
SLC4A5
SLC9A2
SLFN5
SLITRK6
SMARCA2
SMARCD1
SMC5
SNHG9
SNORA40
SNORD3A
SNORD65
SNRK
SNRPB
SNRPB2
SNRPC
SNRPD2
SNX10
SNX16
SNX3
SORT1
SOX3
SOX4
SP110
SPATA7
SPG11
SPIRE1
SPPL2A
SPRR1A
SS18L2
SSR1
SSTR2
STAG2
STK40
STMN3
STRBP
STX7
STXBP5
STYXL1
SUGT1
SULF2
SUPT16H
SVIP
SWAP70
SYNJ2
SYTL1
TACC1
TAGLN2

SLC35B2
SLC35B3
SLC35E1
SLC3A2
SLC7A2
SLC7A6OS
SLK
SMARCD2
SNHG11
SNHG6
SNORD16
SNRNP70
SNX17
SOAT1
SPOP
SPRED2
SQLE
SREBF1
SRP72
SSR4
ST13
ST3GAL1
STXBP3
SURF1
SURF6
SYK
SYNM
TAF10
TAF2
TAF8
TATDN1
TBX2
TC2N
TCEA2
TCEAL4
TCF25
TEAD2
THEX1
THNSL1
TIAF1
TIGA1
TIMM17B
TJP3
TM9SF1
TMED2
TMED5
TMEM121
TMEM132A
TMEM134
TMEM147
TMEM159
TMEM170A
TMEM170B
TMEM184C
TMEM222
TMEM33
TMEM41B
TMEM72
TMX3
TNKS1BP1
TOP2A
TOR1AIP2
TRAPPC10
TRAPPC2L

TAP2
TAPT1
TCF12
TEAD3
TERC
TGFBR2
TGOLN2
THAP10
THBS3
THOC2
TIGD2
TIMM9
TIMP2
TLE1
TM9SF4
TMBIM4
TMC4
TMEFF2
TMEM1
TMEM108
TMEM135
TMEM150A
TMEM189
TMEM43
TMEM64
TMEM79
TMEM83
TMF1
TMTC2
TNNT1
TNS3
TOMM6
TOP1P1
TOP1P2
TOP2B
TPD52L2
TRAK1
TRAPPC3
TRIM24
TRIM4
TRIM8
TRIP12
TRMT61A
TSC22D1
TTC33
TTC39B
TUBB8
TUBD1
TUFT1
UBE2E2
UBE2F
UBE2H
UGDH
UGT1A6
UGT2B7
UHMK1
USHBP1
USP42
USP6NL
VAMP7
VASN
VDAC3
VPS35
VPS54

TRAPPC5
TRAPPC6A
TREML1
TRIM33
TRIM59
TRIP11
TRIP6
TROAP
TSSC1
TSTD1
TTK
TUBA4A
TWF1
TXNIP
UBA1
UBE1C
UBE2D1
UBE2L6
UBE3C
UBXN1
UFC1
UNKL
UPF2
UPK1A
USO1
USP16
USP3
USP5
UTP14C
UXT
VBP1
VCL
VEGFA
VKORC1
VKORC1L1
VPS37D
VPS4A
WBP1
WDR44
WDR61
WDSOF1
WWC3
XRCC5
YPEL3
YWHAB
ZC3H4
ZC3H7A
ZC3HAV1L
ZDHHC4
ZDHHC9
ZFR
ZFYVE16
ZFYVE19
ZNF142
ZNF286C
ZNF317
ZNF490
ZNF503
ZNF581
ZNF611
ZNF621
ZNF770
ZWILCH

WASL
WBP2
WBSCR22
WDR54
WDSUB1
WHAMM
WISP2
WNT8A
WSB2
YES1
YIPF4
YIPF5
YIPF6
YTHDC1
YWHAZ
ZDHHC20
ZDHHC21
ZDHHC7
ZFAND2B
ZFHX3
ZFP90
ZFPL1
ZMIZ2
ZNF140
ZNF16
ZNF217
ZNF322A
ZNF330
ZNF358
ZNF446
ZNF467
ZNF529
ZNF629
ZNF668
ZNF721
ZNF771
ZNF773
ZNF777
ZNF787
ZNF800
ZNF816A
ZNF84
ZPBP
ZWINT
ZYG11B
ZZZ3

Table S4-1. GO Term Enrichment of genes in C1: Constant, diverge-converge

GO term	P_{value}	# genes
GO:0055114~oxidation reduction	0.001001623	10
GO:0005829~cytosol	0.001177962	15
oxidoreductase	0.001811866	9
GO:0006917~induction of apoptosis	0.001827765	7
GO:0012502~induction of programmed cell death	0.001856811	7
GO:0043065~positive regulation of apoptosis	0.007746022	7
GO:0043068~positive regulation of programmed cell death	0.008003318	7
GO:0010942~positive regulation of cell death	0.008178256	7
GO:0008283~cell proliferation	0.008266757	7
GO:0006631~fatty acid metabolic process	0.008486207	5
GO:0042127~regulation of cell proliferation	0.013602753	9
GO:0042981~regulation of apoptosis	0.015313907	9
GO:0043067~regulation of programmed cell death	0.016171712	9
GO:0010941~regulation of cell death	0.016502291	9
GO:0008629~induction of apoptosis by intracellular signals	0.020291167	3
GO:0043232~intracellular non-membrane-bounded organelle	0.020820249	19
GO:0043228~non-membrane-bounded organelle	0.020820249	19
GO:0005198~structural molecule activity	0.022203014	8

Table S4-2. GO Term Enrichment of genes in C2: Constant, concordant

GO term	P_{value}	# genes
phosphoprotein	1.94E-13	367
cytoplasm	1.10E-07	180
GO:0065003~macromolecular complex assembly	1.13E-07	57
GO:0043933~macromolecular complex subunit organization	1.89E-07	59
golgi apparatus	3.58E-07	49
GO:0005829~cytosol	1.62E-06	89
GO:0005794~Golgi apparatus	3.82E-06	64
GO:0031090~organelle membrane	1.13E-05	74
er-golgi transport	1.18E-05	14
GO:0070271~protein complex biogenesis	1.47E-05	42
GO:0006461~protein complex assembly	1.47E-05	42
ubl conjugation	2.30E-05	44
ribonucleoprotein	5.21E-05	26
protein biosynthesis	8.38E-05	20
GO:0016192~vesicle-mediated transport	1.40E-04	43
cross-link:Glycyl lysine isopeptide (Lys-Gly) (interchain with G-Cter in ubiquitin)	1.93E-04	20
GO:0034622~cellular macromolecular complex assembly	2.09E-04	28
GO:0034621~cellular macromolecular complex subunit organization	2.63E-04	30
nucleus	4.24E-04	199
endoplasmic reticulum	4.49E-04	46

Table S4-3. GO Term Enrichment of genes in C3: net change, diverge-converge

GO term	P_{value}	# genes
GO:0030529~ribonucleoprotein complex	6.07E-05	14
GO:0043232~intracellular non-membrane-bounded organelle	9.64E-05	35
GO:0043228~non-membrane-bounded organelle	9.64E-05	35
GO:0005829~cytosol	1.02E-04	23
protein biosynthesis	3.07E-04	8
ribosomal protein	3.07E-04	8
phosphoprotein	3.51E-04	70
ribonucleoprotein	6.58E-04	9
GO:0031974~membrane-enclosed lumen	7.87E-04	26
GO:0005840~ribosome	7.92E-04	8
GO:0006412~translation	8.17E-04	10
GO:0031968~organelle outer membrane	8.23E-04	6
GO:0006414~translational elongation	9.44E-04	6
GO:0019867~outer membrane	9.76E-04	6
GO:0070013~intracellular organelle lumen	0.001014	25
GO:0043233~organelle lumen	0.001398	25
GO:0003735~structural constituent of ribosome	0.001443	7

Table S4-4. GO Term Enrichment of genes in C4: net changed, concordant

GO term	P_{value}	# genes
phosphoprotein	6.47E-14	357
nucleus	4.84E-08	215
GO:0005829~cytosol	1.41E-06	84
GO:0031974~membrane-enclosed lumen	3.60E-05	102
GO:0043233~organelle lumen	7.48E-05	99
GO:0070013~intracellular organelle lumen	8.50E-05	97
GO:0005654~nucleoplasm	1.06E-04	56
GO:0031981~nuclear lumen	1.09E-04	82
GO:0043232~intracellular non-membrane-bounded organelle	1.14E-04	131
GO:0043228~non-membrane-bounded organelle	1.14E-04	131
phosphoric monoester hydrolase	1.70E-04	11
GO:0030532~small nuclear ribonucleoprotein complex	1.96E-04	7
transcription regulation	4.15E-04	102
GO:0003712~transcription cofactor activity	5.55E-04	28
Transcription	6.00E-04	103
actin-binding	6.47E-04	21
alternative splicing	7.79E-04	311
GO:0005794~Golgi apparatus	8.35E-04	52
GO:0005996~monosaccharide metabolic process	8.45E-04	20
GO:0008134~transcription factor binding	8.86E-04	35

Table S5. Breast basal cell and luminal cell gene signatures

up-regulated in Luminal cells vs. Basal (455 genes)	down-regulated in Luminal cells vs. Basal (3801 genes)
ABCD3	ABCA3
ACTN1	ABCG1
ADA	ABHD11
ADD3	ABHD12
ADM	ACVR1B
ADORA2B	ADCY6
ADRB2	AFF3
AGPS	AGR2
AIM1	ANKRD13D
AKR1B1	ANKRD30A
AKR1B10	ANXA6
AKR1C1	ANXA9
AKR1C2	API5
AKR1C3	AR
AKT3	ARF3
ALDH1A3	ARFIP2
ALDH3A2	ARHGEF26
AMD1	ARID2
ANKH	ARRB1
ANKRD33B	ASB8
ANTXR1	ASH1L
ANXA1	ASTN2
ANXA2	ATP2C2
ANXA2P2	ATP6AP1
ANXA3	ATP6V0E2
ANXA4	ATP8B1
APOL6	ATXN7L3B
ARAP3	AVL9
ARHGAP23	BAI2
ARHGAP5	BAZ2A
ARNTL2	BCAS1
ARPC2	BCOR
ASXL1	BLNK
ATP10D	BPTF
ATP1A1	C10orf12
ATP1B3	C10orf82
AXL	C12orf51
B2M	C14orf132
B3GNT5	C17orf28
BICC1	C17orf58
BICD2	C17orf62
BIN1	C19orf46
BIRC3	C4orf19
BMP1	C7orf26
BNC1	C9orf152
BTG3	C9orf7
BTN3A2	C9orf91
C10orf10	CA12
C12orf39	CACNA1D
C13orf15	CACNA2D2
C15orf52	CACNB3
C1R	CACNG4

C1S	CACYBP
C21orf63	CADM1
C21orf96	CAMSAP3
C3	CANT1
C6orf145	CAPN9
C9orf5	CCDC117
CALD1	CCND1
CAMTA1	CDC42SE1
CARD6	CEP350
CASP1	CERS2
CASP4	CERS6
CAV1	CFD
CAV2	CHN2
CBR1	CHTOP
CCDC28A	CIRBP
CCDC80	CISH
CCDC82	CLSTN2
CCDC88A	CREB3L1
CCNA1	CREB3L4
CCNYL1	CRNKL1
CD109	CSNK1D
CD14	CSRNP2
CD44	CTNND2
CD58	CTXN1
CD59	CXXC5
CD97	CYB561
CDC42EP3	CYHR1
CDCP1	DAAM1
CDH13	DACH1
CDH3	DDAH2
CDK6	DDX42
CEBPD	DEGS2
CFB	DENND1A
CFI	DENND4B
CFL2	DEPTOR
CFLAR	DHRS13
CHMP1B	DIP2C
CHST3	DKFZP586I1420
CLIC4	DLG3
CLIP4	DNAJA4
CLMP	DNAJC1
CMPK1	DNALI1
COL4A1	DOPEY2
COL4A2	DSCAM-AS1
COL8A1	DUSP8
COPS8	EFR3B
CORO1C	EIF3B
COTL1	ELOVL2
CRIPT	EMP2
CRK	ENPP1
CRYAB	EPN3
CSDA	EPS8L1
CSNK2A2	ERBB3
CTSC	ERGIC1
CXCL1	ESR1
CXCL2	ESRP2
CXCL3	ETNK2
CYB5R3	EVL
CYBRD1	F7
CYLD	FAM110B
DCBLD1	FAM115A
DCBLD2	FAM46C
DCTD	FAM65C
DGKA	FBRSL1

DIRC2	FGFR4
DMD	FKBP4
DNAJB4	FLJ22184
DOCK5	FLJ38379
DPYD	FLJ45983
DSC3	FOXA1
DSE	FRMD4A
DSG2	FRS2
DSG3	FTX
DST	FUS
DUOX1	FZD4
EGFR	GALNT6
EHBP1	GAMT
ELK3	GARNL3
ELL2	GARS
EMP1	GART
EMP3	GATA3
EPHA2	GGA1
ERAP2	GGA3
EREG	GNA12
ESYT2	GOLT1A
ETF1	GPD1L
ETS1	GPR160
ETS2	GPRC5C
EXT1	GRAMD4
F2RL1	GSPT1
F3	GTF3C1
FAM101B	HEXDC
FAM171A1	HIP1R
FAM176A	HK2
FAM69A	HMG20B
FAM83A	HMGCS2
FAM83D	HNRNPA2B1
FAM92A1	HPN
FAP	HPX
FAS	ICA1
FBLIM1	INHBB
FDFT1	INPP5J
FERMT1	INTS3
FGF2	IQCE
FGFBP1	IQSEC1
FHL1	IRGQ
FKBP1A	ISG20
FMNL2	IVD
FNDC3B	JHDM1D
FOSL1	KAT6B
FOXQ1	KCTD15
FRMD6	KDM4B
FSCN1	KIAA0040
FST	KIAA0182
FSTL1	KIAA0226
FXYD5	KIAA0232
FZD6	KIAA0556
GABRE	KIAA0889
GALNT2	KIAA0913
GART	KIAA1211
GAS1	KIAA1244
GBP1	KIAA1324
GBP3	KIAA1467
GFOD1	KIAA1598
GJB3	KIF12
GJC1	KIFC2
GLIPR1	KLF2
GM2A	KLHDC9

GNA15	KLHL22
GNAI1	KLRG2
GNAL	KRT19
GNG12	LARGE
GPM6B	LARP4B
GPSM2	LFNG
GPX1	LINC00312
GPX8	LLGL2
GSTP1	LMCD1
GTF2B	LNX1
HIF1A	LOC100130987
HLA-E	LOC100272216
HMGA2	LOC100506966
HOXA1	LOC643837
HOXA3	LOC692247
HOXA5	LONRF2
HP1BP3	LRP3
HRCT1	LRRN1
HRH1	LUC7L3
HSD17B11	LZTR1
HTRA1	MAGED2
ICAM1	MAPK9
IFI16	MAPT
IFI27	MARS
IFI44	MB21D2
IFIT3	MCCC2
IFNGR1	MDM4
IGF2BP2	MEGF9
IGF2BP3	MGAT4A
IGFBP6	MGRN1
IGFBP7	MIF4GD
IL15	MLL2
IL18	MLPH
IL1A	MTERFD3
IL1RAP	MXRA8
IL20RB	MYB
IL7R	MYCN
INHBA	MYEF2
INPP1	MYO5B
IRS2	MYO6
IRX1	NACA
ITGA3	NBPF1
ITGA6	NDUFS8
ITGB1	NKAIN1
ITGB8	NLK
ITM2C	NME3
JAG1	NPDC1
KIF1B	NUCB2
KIRREL	NUDT4
KLF5	ONECUT2
KLHL29	P4HTM
KLK10	PATZ1
KLK5	PBX1
KPNA1	PCBP2
KRT14	PCK2
KRT15	PCP4
KRT16	PGGT1B
KRT17	PGR
KRT5	PI4KA
KRT6A	PLA2G12A
KRT6B	PLCXD1
LAMA3	PLEKHH1
LAMB3	PLXNA3
LAMC1	POGZ

LAMC2	POLE
LARP6	POMT1
LBH	PPP1R16A
LEPREL1	PPP2R2C
LIPG	PREX1
LOC100499467	PRLR
LOC100505633	PRR14
LOC100506621	PRRC2C
LOC285812	PRRT2
LOC346887	PRRT3
LOX	PTPRF
LOXL2	PVRL2
LUZP1	PYCR1
LY6K	RAB11FIP3
LYN	RAB17
MAML2	RAB3D
MAP4K4	RAB40C
MAP7D3	RABEP2
MBNL1	RALGAPA1
MBNL2	RALGPS1
MBP	RBAK
MDFIC	RDH13
MDH1	REEP5
MET	RGL2
MFGE8	RHBDF1
MICALL1	RHOB
MIR100HG	RHOH
MIR22HG	RHPN1
MIR31HG	RIIAD1
MMADHC	RND1
MME	RNF103
MMP14	RSAD1
MPZL1	RSPH1
MSN	RUSC1
MT1E	SBK1
MT1F	SCUBE2
MT1G	SCYL3
MT1H	SDCCAG3
MT1P2	SEC16A
MT1X	SECISBP2
MT2A	SERF2
MTMR2	SFI1
MYL12A	SFMBT2
MYO1B	SH3GLB2
MYO1E	SHANK2
NAB1	SIDT1
NAMPT	SIDT2
NAV2	SLC16A6
NCK1	SLC1A4
NDEL1	SLC24A3
NDFIP2	SLC25A29
NFAT5	SLC25A44
NFE2L2	SLC26A11
NMI	SLC2A10
NNMT	SLC35A1
NOB1	SLC37A1
NR3C1	SLC38A1
NRP1	SLC38A10
NSFL1C	SLC44A4
NT5E	SLC4A8
NUDT15	SLC7A8
NUP50	SLC9A3R1
NXN	SMARCC2
OBFC2A	SNED1

OGFRL1	SNX27
ORMDL1	SOX12
OSBPL3	SOX13
OSBPL9	SPATA2L
OSMR	SPDEF
PARP4	SPTLC2
PDGFC	SRRM2
PDP1	STARD10
PDZK1IP1	STRADA
PELO	STRBP
PERP	SYCP2
PGM2	SYNGR2
PHLDA1	TADA2B
PHLDB2	TAPT1
PI3	TBC1D16
PIK3CD	TBC1D30
PKN2	TBL1X
PKP2	TBX3
PLA2G4A	TC2N
PLAT	TESK1
PLAU	TFF1
PLS3	TFF3
PLSCR1	TGFB3
PM20D2	TGIF2
PNLIPRP3	THSD4
PPP1R14C	THUMPD1
PPP4R1	TJP3
PRKCDBP	TLE3
PRKD3	TMBIM6
PRNP	TMEM150C
PRSS12	TMEM184A
PSAT1	TMEM229B
PSMB8	TMEM57
PSMB9	TMEM80
PTGS2	TNIP1
PTK7	TNRC18
PTPN2	TOB1
PTPRM	TOMM70A
PTRF	TRAPPC9
RAC2	TRIL
RALB	TRIM3
RALBP1	TRPS1
RBFOX2	TSPAN13
RBM7	TSPAN15
RBMS1	TTC3
RBMS3	TTC39A
RETSAT	TTC9
REXO2	UAP1L1
RGL1	UBN1
RGNEF	ULK1
RGS2	USP3
RGS20	USP42
RIOK3	USP7
RIPK4	VIPR1
RND3	VPS37C
RNF145	VPS72
RRAS2	WFS1
RUNX3	XBP1
S100A10	ZBTB42
S100A2	ZDHHC8P1
SAMD9	ZFYVE16
SCHIP1	ZMIZ1
SCPEP1	ZNF12
SEL1L3	ZNF24

SEP15	ZNF296
SEP10	ZNF398
SERPINB2	ZNF444
SERPINB5	ZNF467
SERPINE2	ZNF703
SFN	ZNF704
SFRP1	ZNF74
SGK1	ZNF84
SH3D19	
SH3GLB1	
SH3KBP1	
SIRPA	
SKAP2	
SLC16A1	
SLC1A3	
SLC25A37	
SLC6A15	
SLC9A6	
SLPI	
SMAD3	
SMCHD1	
SNAI2	
SNX7	
SOAT1	
SOX7	
SP100	
SPATS2L	
SPRY2	
SPTBN1	
SRI	
SRPX	
SRPX2	
SSFA2	
STAMBPL1	
STAT3	
STAT4	
STK17A	
SVIL	
TAP2	
TAX1BP3	
TBC1D1	
TBPL1	
TGFA	
TGFBI	
TGFBR2	
TKT	
TLE4	
TLR2	
TM2D1	
TMED5	
TMEM154	
TMEM173	
TMEM30A	
TNFAIP3	
TNFAIP8	
TNFRSF10D	
TOX2	
TP63	
TRIM22	
TRIM29	
TRIP10	
TRMT6	
TUBA4A	
TUBB6	

TWIST2
TWSG1
UBASH3B
UBE2E3
UPP1
VAMP3
VSNL1
WBP5
WDR1
WLS
WWTR1
YAP1
YBX1
YES1
ZBTB16
ZBTB38
ZC3H12C
ZDHHC2
ZMYM6

Table S6-1. Gene set enrichment analysis (GSEA) Day 1 up-regulated pathways vs. Day 0

Gene set	Size	NES	NOM p-val
KEGG_METABOLISM_OF_XENOBIOTICS_BY_CYTOCHROME_P450	30	1.9015	0
KEGG_RETINOL_METABOLISM	19	1.7918	0.001862
KEGG_STEROID_HORMONE_BIOSYNTHESIS	16	1.758	0
Stress Response	33	1.6812	0
Stemness Markers	22	1.6572	0.003759
REACTOME_BIOLOGICAL_OXIDATIONS	54	1.5728	0.001901
TURASHVILI_BREAST_DUCTAL_CARCINOMA_VS_LOBULAR_NORMAL_UP	49	1.5493	0.005435
PID_INTEGRIN3_PATHWAY	21	1.5448	0.01495
KEGG_TRYPTOPHAN_METABOLISM	24	1.5363	0.01132
REACTOME_PPARA_ACTIVATES_GENE_EXPRESSION	74	1.5246	0.007722
PID_WNT_NONCANONICAL_PATHWAY	24	1.4902	0.01845
REACTOME_FATTY_ACID_TRIACYLGLYCEROL_METABOLISM	124	1.4818	0.01512
TURASHVILI_BREAST_NORMAL_DUCTAL_VS_LOBULAR_UP	45	1.4769	0.02239
KEGG_PORPHYRIN_AND_CHLOROPHYLL_METABOLISM	24	1.4691	0.04656
PID_ERBB4_PATHWAY	27	1.4494	0.04934
TURASHVILI_BREAST_LOBULAR_CARCINOMA_VS_LOBULAR_NORMAL_UP	51	1.4255	0.02186
KEGG_O_GLYCAN_BIOSYNTHESIS	15	1.4137	0.06814
BIOCARTA_IGF1_PATHWAY	15	1.4104	0.05311
BIOCARTA_INSULIN_PATHWAY	16	1.3738	0.06897
Prolactin signaling pathway	43	0.62659	0.9497

Table S6-2. Gene set enrichment analysis (GSEA) Day 1 down-regulated pathways vs. Day 0

Gene set	Size	NES	NOM p-val
REACTOME_INTERFERON_GAMMA_SIGNALING	37	1.7986	0.001984
REACTOME_INTERFERON_SIGNALING	111	1.7965	0
REACTOME_CYTOKINE_SIGNALING_IN_IMMUNE_SYSTEM	179	1.7637	0
REACTOME_INTERFERON_ALPHA_BETA_SIGNALING	41	1.7234	0.001972
KEGG_FRUCTOSE_AND_MANNOSE_METABOLISM	28	1.5556	0.01359
REACTOME_GLUCOSE_METABOLISM	52	1.5539	0.009766
REACTOME_METABOLISM_OF_CARBOHYDRATES	152	1.5428	0.001942
REACTOME_IMMUNOREGULATORY_INTERACTIONS_LYMPHOID_NON_LYMPHOID_CELL	23	1.5419	0.02299
REACTOME_GLYCOLYSIS	22	1.5296	0.01357
REACTOME_NEGATIVE_REGULATORS_OF_RIG_I_MDA5_SIGNALING	23	1.5176	0.04016
BIOCARTA_MCALPAIN_PATHWAY	17	1.512	0.02806
PID_MYC_REPRESSPATHWAY	51	1.5075	0.01461
HUPER_BREAST_BASAL_VS_LUMINAL_UP	29	1.4952	0.01047
PID_IL12_2PATHWAY	30	1.4814	0.03346
KEGG_SPLICEOSOME	104	1.4716	0.01553
REACTOME_GLUCONEOGENESIS	24	1.4512	0.03536
REACTOME_RESPIRATORY_ELECTRON_TRANSPORT	61	1.4106	0.1008
REACTOME_RNA_POL_I_TRANSCRIPTION	60	1.4082	0.03929

Table S6-3. Gene set enrichment analysis (GSEA) Day 5 up-regulated pathways vs. Day 0

Gene set	Size	NES	NOM p-val
KEGG_TRYPTOPHAN_METABOLISM	24	-1.6197	0.005859
BIOCARTA_ALK_PATHWAY	21	-1.5426	0.01613
PID_ERBB4_PATHWAY	27	-1.5418	0.008264
REACTOME_SYNTHESIS_OF_PIPS_AT_THE_PLASMA_MEMBRANE	24	-1.5	0.0409
REACTOME_SIGNALING_BY_HIPPO	15	-1.4979	0.06962
BIOCARTA_RAS_PATHWAY	22	-1.4779	0.04065
KEGG_PORPHYRIN_AND_CHLOROPHYLL_METABOLISM	24	-1.4294	0.06751
TURASHVILI_BREAST_NORMAL_DUCTAL_VS_LOBULAR_UP	45	1.421	0.04286
PID_PS1PATHWAY	35	-1.4208	0.04732
BIOCARTA_WNT_PATHWAY	21	-1.4131	0.04277
REACTOME_SIGNALING_BY_RHO_GTPASES	66	-1.4082	0.04848
KEGG_PHOSPHATIDYLINOSITOL_SIGNALING_SYSTEM	52	-1.3904	0.04878
KEGG_STEROID_HORMONE_BIOSYNTHESIS	16	-1.3848	0.0846
Stress Response	33	-1.3769	0.1175
BIOCARTA_IGF1_PATHWAY	15	-1.3701	0.08299
BIOCARTA_INSULIN_PATHWAY	16	-1.358	0.07724
REACTOME_TRANSCRIPTIONAL_REGULATION_OF_WHITE_ADIPOCYTE_DIFFERENTIATION	54	1.2819	0.1223
Prolactin signaling pathway	43	-1.207	0.233

Table S6-4. Gene set enrichment analysis (GSEA) Day 5 down-regulated pathways vs. Day 0

Gene set	Size	NES	NOM p-val
HUPER_BREAST_BASAL_VS_LUMINAL_UP	29	-1.5767	0.001992
Epithelial Signature Genes	31	1.5497	0.02505
REACTOME_INTERFERON_ALPHA_BETA_SIGNALING	41	1.5494	0.025
PID_MYC_REPRESSPATHWAY	51	1.5226	0.01663
REACTOME_S_PHASE	93	1.5045	0.0121
REACTOME_INTERFERON_SIGNALING	111	1.4954	0.05979
KEGG_DNA_REPLICATION	35	1.4902	0.02434
REACTOME_DNA_STRAND_ELONGATION	29	1.4734	0.02899
REACTOME_ASSEMBLY_OF_THE_PRE_REPLICATIVE_COMPLEX	52	1.4701	0.04546
REACTOME_ORC1_REMOVAL_FROM_CHROMATIN	53	1.4698	0.03666
KEGG_COMPLEMENT_AND_COAGULATION_CASCADES	16	1.4689	0.02053
REACTOME_SYNTHESIS_OF_DNA	77	1.4655	0.03106
REACTOME_G1_S_TRANSITION	89	1.4653	0.03279
REACTOME_GLUCOSE_METABOLISM	52	1.4652	0.02245
REACTOME_METABOLISM_OF_MRNA	191	1.4567	0.0167
REACTOME_M_G1_TRANSITION	64	1.4558	0.05405
REACTOME_SCFSKP2_MEDIATED_DEGRADATION_OF_P27_P21	49	1.4545	0.04481
REACTOME_ER_PHAGOSOME_PATHWAY	52	1.453	0.05955
REACTOME_G2_M_CHECKPOINTS	30	1.4457	0.064
KEGG_MISMATCH_REPAIR	22	1.4447	0.04508
REACTOME_CYTOKINE_SIGNALING_IN_IMMUNE_SYSTEM	179	1.4424	0.05589

Table S7. Candidate genes identified from CAP-Net analysis which cause MCF7 cell to differentiate

Gene	Biological functions	Effects	Reference
IFI27	A protein that promotes cell death and mediates IFN-induced apoptosis, characterized by a rapid and robust release of cytochrome C from the	Inhibition	[2,3]
VEGFA	A growth factor that regulates angiogenesis. The CAP-Net shows a low expression of VEGFA, which indicates a reduction in the angiogenesis and tumor	Inhibition	[4–6]
CSF1	A cytokine that enhances the tumor growth via tumor-associated macrophages. It is highly expressed in several subtypes of breast cancer and causes the macrophage differentiation by stimulating VEGFA. The down-regulation of CSF1	Inhibition	[7–9]
AKT1	A Serine-Threonine protein kinase that regulates metabolism, proliferation, cell survival, growth and angiogenesis	-	[10]
IL8	A chemotactic factor that attracts neutrophils, basophils and T-cells. It activates neutrophils. It is released from several cell types in response to an inflammatory	inhibition	[11]
CYP1B1	An enzyme that metabolizes drugs, involved in electron transport pathway.	activation	[12]
DHCR24	An enzyme that protects cells from apoptosis induced by oxidative stress	activation	[13,14]
EDN1	A protein that is used to produce vasoconstrictive peptides	activation	[15]
HMOX1	An enzyme that cleaves the heme ring at the alpha methene bridge to form [illegible]	activation	[16]
MAOA	A mitochondrial enzyme that degrades monoamines neuron transmitters and dietary amines, induces EMT through activation of VEGF	Inhibition	[17,18]
MUC1	A membrane protein that activated T-cells, influences the Ras/MAPK pathway, promotes tumor progression, regulates TP53-mediated transcription, determines	Inhibition	[19–21]

SUPPLEMENTAL REFERENCES

1. Tsuchiya M, Piras V, Giuliani A, Tomita M, Selvarajoo K. Collective dynamics of specific gene ensembles crucial for neutrophil differentiation: the existence of genome vehicles revealed. PLoS ONE. 2010;5:e12116.

2. Cheriyath V, Leaman DW, Borden EC. Emerging roles of FAM14 family members (G1P3/ISG 6-16 and ISG12/IFI27) in innate immunity and cancer. Journal of interferon & cytokine research. 2011;31:173–181.

3. Suomela S, Cao L, Bowcock A, Saarialho-Kere U. Interferon alpha-inducible protein 27 (IFI27) is upregulated in psoriatic skin and certain epithelial cancers. Journal of Investigative Dermatology. 2004;122:717–721.

4. Goel H, Mercurio A. VEGF targets the tumour cell. Nature reviews Cancer. 2013;13:871–882.

5. Bender RJ, Gabhann} F, Mac Gabhann F. Expression of VEGF and semaphorin genes define subgroups of triple negative breast cancer. PLoS ONE. 2013;8:e61788.

6. Pathak AP, McNutt S, Shah T, Wildes F, Raman V, Bhujwalla ZM. In vivo "MRI phenotyping" reveals changes in extracellular matrix transport and vascularization that mediate VEGF-driven increase in breast cancer metastasis. PLoS ONE. 2013;8:e63146.

7. Xu J, Escamilla J, Mok S, David J, Priceman S, West B, Bollag G, McBride W, Wu L. CSF1R signaling blockade stanches tumor-infiltrating myeloid cells and improves the efficacy of radiotherapy in prostate cancer. Cancer research. 2013;73:2782–94.

8. Strachan DC, Ruffell B, Oei Y, Bissell MJ, Coussens LM, Pryer N, Daniel D. CSF1R inhibition delays cervical and mammary tumor growth in murine models by attenuating the turnover of tumor-associated macrophages and enhancing infiltration by CD8(+) T cells. Oncoimmunology. 2013;2:e26968.

9. Richardsen E, Uglehus RD, Johnsen SH, Busund L-T. Macrophage-Colony Stimulating Factor (CSF1) Predicts Breast Cancer Progression and Mortality. Anticancer research. 2015;35:865–74.

10. Domingo-Domenech J, Vidal SJ, Rodriguez-Bravo V, Castillo-Martin M, Quinn SA, Rodriguez-Barrueco R, Bonal DM, Charytonowicz E, Gladoun N, de la Iglesia-Vicente J, et al. Suppression of Acquired Docetaxel Resistance in Prostate Cancer through Depletion of Notch- and Hedgehog-Dependent Tumor-Initiating Cells. Cancer cell. 2012;22:373–88.

11. Kim S, Goel S, Alexander CM. Differentiation Generates Paracrine Cell Pairs That Maintain Basaloid Mouse Mammary Tumors: Proof of Concept Oshima R, editor. PLoS ONE. 2011;6:e19310.

12. Faiq MA, Dada R, Sharma R, Saluja D, Dada T. CYP1B1: A Unique Gene with Unique Characteristics. Current drug metabolism. 2014;15:893–914.

13. Lee GT, Ha Y-S, Jung YS, Moon S-K, Kang HW, Lee O-J, Joung JY, Choi YH, Yun S-J, Kim W-J, et al. DHCR24 is an Independent Predictor of Progression in Patients with Non-Muscle-Invasive Urothelial Carcinoma, and Its Functional Role is Involved in the Aggressive Properties of Urothelial Carcinoma Cells. Annals of Surgical Oncology. 2014;21:538–545.

14. Battista M-C, Guimond M-O, Roberge C, Doueik AA, Fazli L, Gleave M, Sabbagh R, Gallo-Payet N. Inhibition of DHCR24/seladin-1 impairs cellular homeostasis in prostate cancer. Prostate. 2010;70:921–933.

15. Lu J-W, Liao C-Y, Yang W-Y, Lin Y-M, Jin S-LC, Wang H-D, Yuh C-H. Overexpression of endothelin 1 triggers hepatocarcinogenesis in zebrafish and promotes cell proliferation and migration through the AKT pathway. PLoS ONE. 2014;9:e85318.

16. Andrés NC, Fermento ME, Gandini NA, Romero AL, Ferro A, Donna LG, Curino AC, Facchinetti MM. Heme oxygenase-1 has antitumoral effects in colorectal cancer: involvement of p53. Experimental and molecular pathology. 2014;97:321–31.

17. White TA, Kwon EM, Fu R, Lucas JM, Ostrander EA, Stanford JL, Nelson PS. The Monoamine Oxidase A gene promoter repeat and prostate cancer risk. Prostate. 2012;72:1622–1627.

18. Wu JB, Shao C, Li X, Li Q, Hu P, Shi C, Li Y, Chen Y-T, Yin F, Liao C-P, et al. Monoamine oxidase A mediates prostate tumorigenesis and cancer metastasis. Journal of Clinical Investigation. 2014;124:2891–2908.

19. Taylor-Papadimitriou J, Burchell J, Miles DW, Dalziel M. MUC1 and cancer. Biochimica et Biophysica Acta - Molecular Basis of Disease. 1999;1455:301–313.

20. Zaretsky JZ, Barnea I, Aylon Y, Gorivodsky M, Wreschner DH, Keydar I. MUC1 gene overexpressed in breast cancer: structure and transcriptional activity of the MUC1 promoter and role of estrogen receptor alpha (ERalpha) in regulation of the MUC1 gene expression. Molecular cancer. 2006;5:57.

21. Nath S, Mukherjee P. MUC1: A multifaceted oncoprotein with a key role in cancer progression. Trends in Molecular Medicine. 2014;20:332–342.
